# Aryl hydrocarbon receptor pharmacology—mechanisms, ligands, and therapeutic potential

**DOI:** 10.1016/j.pharmr.2026.100146

**Published:** 2026-06-03

**Authors:** Raitis Bobrovs, Ninni Elise Olafsen, Samaneh Shabani Åhrling, Jason Matthews

**Affiliations:** 1 Latvian Institute of Organic Synthesis, Riga, Latvia; 2 Department of Nutrition, Institute of Basic Medical Sciences, Faculty of Medicine, University of Oslo, Oslo, Norway; 3 Department of Pharmacology and Toxicology, University of Toronto, Toronto, Ontario, Canada

## Abstract

The aryl hydrocarbon receptor (AHR) is a ligand activated transcription factor that has emerged as a key modulator of several physiological and pathological processes. Historically, AHR has been studied for its role as a mediator of the toxic responses of environmental pollutants, such as 2,3,7,8-tetrachlorodibenzo-*p*-dioxin. Because of this, its potential as a therapeutic target was overlooked. AHR is now regarded as a multifunctional regulator of inflammation, immunity, barrier tissue integrity, metabolism, and cancer biology. AHR signaling is modulated by an array of structurally diverse endogenous, microbial, dietary, pharmaceutical, and xenobiotic ligands. AHR exhibits extensive crosstalk with many signaling pathways, which contributes to highly context-specific biological outcomes. Recent advances and enhanced interest in AHR pharmacology have accelerated the development of therapeutically relevant AHR ligands, including the clinically approved AHR agonist, tapinarof, for the treatment of psoriasis and atopic dermatitis, as well as emerging AHR antagonist strategies in cancer immunotherapy. Structural efforts have succeeded in providing valuable insight into ligand-AHR interactions that will further contribute to improved and rational design of AHR ligands. However, significant challenges remain, including AHR ligand promiscuity, complex negative feedback regulation, extensive signaling crosstalk, and long-term safety concerns. Here, we review the molecular mechanisms of AHR activation, its physiological and pathological functions, and the current advances of its therapeutic targeting.

**Significance Statement:**

The aryl hydrocarbon receptor (AHR) has emerged from its origins in toxicology to become a promising clinically relevant target for the treatment of dermatological, gastroenterological, and autoimmune diseases as well as cancer. This review summarizes the current knowledge of AHR pharmacology, including molecular signaling, endogenous and exogenous ligands, physiological and pathological functions, present and emerging therapeutic targeting strategies, as well as challenges that remain for translation into safe clinical therapies.

## I Introduction

The aryl hydrocarbon receptor (AHR) is a ligand-activated transcription factor that has been historically studied because of its role in mediating the toxic effects of environmental pollutants, including polycyclic aromatic hydrocarbons (PAHs) and halogenated aromatic hydrocarbons (HAHs), such as 2,3,7,8-tetrachlorodibenzo-*p*-dioxin (TCDD).^1^^,^^2^ Once viewed primarily as a xenobiotic sensor, AHR is now recognized as an evolutionarily conserved ancient regulator of immune responses, inflammation, epithelial barrier integrity, host-microbiome communication, metabolism, differentiation, and proliferation. This diverse physiological profile has transformed AHR from a toxicological receptor into a promising yet complex therapeutic target for dermatological, gastroenterological, and autoimmune diseases as well as cancer. AHR’s role in cancer is, however, multifaceted as it can act as both a tumor suppressor and tumor promoter in a cancer type-dependent manner.^3^^,^^4^

**Fig. 1:**
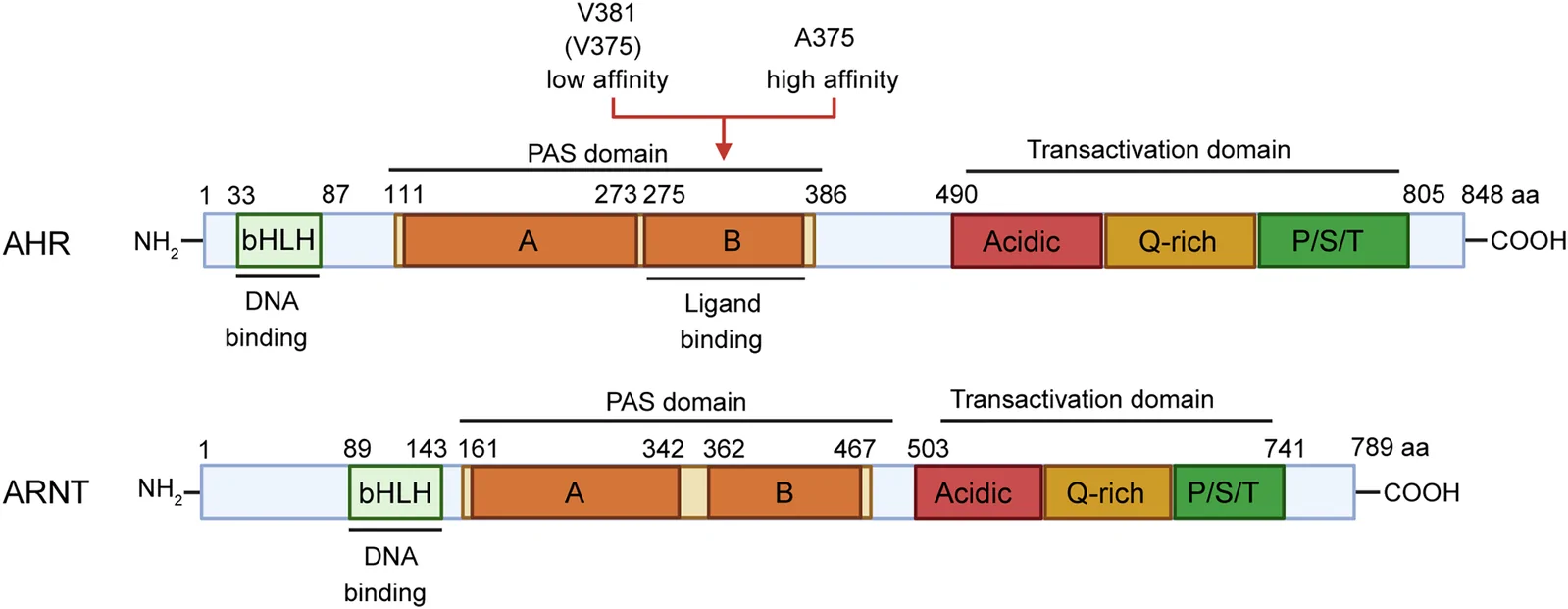
Domain architecture of human AHR and ARNT proteins. The numbers above the domain boundaries refer to the amino acid residues in each protein. The red arrow highlights V381 in human AHR and V375 or A375 corresponding to mouse *Ahr*^*d*^ and *Ahr*^*b1*^ alleles, respectively. Q-rich, glutamine rich; P/S/T, proline/serine/threonine rich; aa, amino acid. Created in BioRender. Matthews, J. (2026) https://BioRender.com/wv1mna0.

AHR is a member of the basic helix-loop-helix/period-aryl hydrocarbon receptor nuclear translocator (ARNT)-single minded (bHLH-PAS) family of transcription factors. The bHLH-PAS proteins represent a diverse family that regulates a spectrum of physiological and pathological responses, including circadian rhythm, oxygen homeostasis, energy metabolism, development, neurological disorders, xenobiotic/drug metabolism, and cancer.^5^ In addition to AHR, ARNT (also known as hypoxia factor 1*β* [HIF1B]), and aryl hydrocarbon receptor repressor (AHRR) are also members of the bHLH-PAS family, and together they regulate numerous cellular and physiological responses, including drug metabolism, immunity, inflammation, barrier integrity, and proliferation.^6^^,^^7^ Other family members include hypoxia-inducible factors (hypoxia factor 1α [HIF1A], HIF2A, and HIF3A) that together with HIF1B, react to hypoxia and stimulate glycolysis and angiogenesis. Circadian locomotor output cycles kaput regulator and the brain and muscle ARNT-like 1 and 2 proteins regulate circadian rhythm. Single-minded 1 and 2, neural PAS domain proteins are essential for neuronal development, whereas nuclear receptor coactivators 1 (NCoA1), NCoA2, and NCoA3 modulate gene expression levels.^8^^,^^9^ Although some bHLH-PAS family members are activated by endogenous stimuli, AHR is one of the few family members that exhibits ligand binding. The bHLH domain of AHR contains a nuclear localization sequence, whereas the PAS domains harbor chaperone protein binding regions with the PAS-B domain also containing the ligand binding domain (Fig. 1).^10^^,^^11^ Ligand binding has also been described for HIF2A,^12^^,^^13^ ARNT,^14^ and Methoprene-tolerant protein, an insect-specific bHLH-PAS protein that is responsive to a sesquiterpenoid (juvenile hormone) important for insect development.^15^^,^^16^ The tripartite C-terminal transactivation domain of AHR consists of acidic, glutamine-rich, proline/serine/threonine-rich sequences that contribute to the magnitude of AHR-dependent changes in gene expression through interaction with coregulator proteins.^17^

AHR displays broad ligand promiscuity, and its activity is modulated by several structurally diverse compounds from the diet, endogenous metabolism, commensal microbe metabolism, environmental pollutants, and pharmaceutical agents. As the range of identified exogenous, xenobiotic and endogenous ligands expands, AHR is increasingly recognized as a fundamental and irreplaceable regulator of normal physiology. However, depending on ligand type, dose, metabolic stability, and cellular context, AHR activation can produce immunostimulatory, immunosuppressive, proliferative, antiproliferative, or toxic effects.^3^^,^^18^, ^19^, ^20^ Understanding this complex and context-dependent signaling is essential to exploit the therapeutic potential of AHR. This review synthesizes current knowledge of AHR pharmacology, including molecular signaling, endogenous and exogenous ligands, physiological and pathological functions, present and emerging therapeutic targeting strategies, as well as challenges that remain for translation into safe clinical therapies.

## II Aryl hydrocarbon receptor

### A Discovery of aryl hydrocarbon receptor

The discovery of AHR resulted from early work in the 1950s studying the role of the PAHs, benzo[*a*]pyrene (B[*a*]P) and 3-methylcholanthrene in the induction of aryl hydrocarbon hydroxylase (AHH) activity.^21^^,^^22^ AHH activity was later linked to cytochrome P450 1 (CYP1) enzyme activity, which includes cytochromes P450 1A1 (CYP1A1), CYP1A2, and CYP1B1. CYP1 enzymes play key roles in the metabolic activation and detoxification, but also in the toxicity of several PAHs and aromatic amines; many of which are CYP1 substrates and AHR ligands.^23^ It is now known that all 3 CYP1 enzymes are regulated by AHR. Before the identification and cloning of AHR, differences in the sensitivity between and within different species to PAH and HAH toxicities gave the first clues for a common genetic component regulating this effect.

The crossing and backcrossing of several different mouse strains led to the identification of what was termed the *Ah* (aryl hydrocarbon) locus, which was suspected to regulate AHH activity.^24^ The *Ah* locus (now referred to as *Ahr* locus) segregated as an autosomal dominant trait in crosses between commonly used C57BL/6 × DBA/2 mouse strains, where the dominant “responsive” allele was named *Ahr*^*b*^ (known now as *Ahr*^*b1*^) in C57BL/6 strains, whereas the “nonresponsive” allele found in the DBA/2J strain was named *Ahr*^*d*^. The “nonresponsive” mouse strains refer to the inability of high dose of nonhalogenated PAHs to induce CYP1 enzyme activity.^25^ However, the finding that “nonresponsive” strains responded to TCDD, a more potent CYP1 enzyme inducer, led to the hypotheses that the *Ahr* locus encodes a receptor protein that controls AHH (CYP1) induction, and that a mutation in this receptor protein would reduce its affinity for ligands in “nonresponsive” strains.^2^ These were later confirmed after the human and mouse *AHR* gene sequences were determined.^26^, ^27^, ^28^ The human *AHR* gene is located on chromosome 7p16.9-17.3 and contains 11 exons that code for the AHR protein of 848 amino acids with a molecular weight of 96 kDa. The mouse *Ahr* gene is located on chromosome 12:35.4–35.5 and contains 11 exons. A comparison between the AHR sequences from C57BL/6 (*Ahr*^*b1*^; predicted protein molecular weight 95 kDa) and DBA/2 (*Ahr*^*d*^; 104 kDa) strains revealed an alanine to valine substitution at position 375 in C57BL/6 AHR and an elongated carboxyl terminus that resulted from mutated stop codon in the *Ahr*^*d*^ allele with an approximate 10-fold lower affinity for [^3^H]TCDD as well as reduced sensitivity to PAH and HAH toxicities. In addition to *Ahr*^*b1*^ and *Ahr*^*d*^, 2 other *Ahr* alleles referred to as *Ahr*^*b2*^ (104 kDa) and *Ahr*^*b3*^ (105 kDa) have been described.^29^ The 3 *Ahr*^*b*^ alleles have ∼10-times higher ligand affinity for TCDD than the *Ahr*^*d*^ allele.^30^ The expression of *Ahr* alleles varies among commonly used laboratory inbred strains (Table 1).^31^^,^^32^ The choice of mouse strain affects AHR-mediated responses under different experimental conditions.^33^ Moreover, the widely used *Ahr* conditional null mice harbor the low-affinity *Ahr*^*d*^ allele because of the use of 129sv strain in their generation.^34^ Gene editing was used to introduce a V375A polymorphism to convert the low-affinity allele into a high-affinity allele, offering a more sensitive *Ahr* conditional model.^35^ Human AHR contains valine in the corresponding 381 amino acid position, resulting in a lower affinity for TCDD compared with products of the mouse *Ahr*^*b*^ alleles.^36^

**Table 1:** *Ahr* alleles found in different laboratory mouse strains compared with human *AHR*

Allele	Mouse Strain	Affinity for TCDD	AA Sequence Length	MW (kDa)
*Ahrb**^1^*	C57BL/6, C58, MA/My, BXD100, BXD91	High (A375)	805	95
*Ahr*^*b2*^	BALB/cBy, A/J, C3H, FVB, CBA	High (A375)	848	104
*Ahr*^*b3*^	MOLF/Ei, *Mus caroli, Mus spretus*	High (A375)	883	105
*Ahr*^*d*^	AKR, DBA/2, 129Sv, SWR, RF, NZB, BXD40, NOD/ShiLtJ	Low (V375)	848	104
*AHR*	Human	Low (V381)	848	104

AA, anthranilic acid; MW, molecular weight.

The cloning of mouse *Ahr* gene led to the generation of *Ahr* deficient mice by 3 different research groups.^37^, ^38^, ^39^ Although some phenotypes differ among the 3 *Ahr* knockout mice*,* all of them are insensitive to the induction of AHR-responsive genes in response to TCDD and resistant to the dioxin toxicities, including thymic atrophy, immunotoxicity, hepatotoxicity, carcinogenicity, teratogenesis, and lethal wasting syndrome.^40^^,^^41^ Studies of *Ahr* knockout mice provided unequivocal proof for the role of AHR in dioxin toxicity and xenobiotic metabolism, but they also revealed an essential role of AHR in normal physiology. *Ahr*^*−/−*^ mice exhibit incomplete closure of ductus venosus, hepatic fibrosis, cardiac hypertrophy, altered immunity, decreased barrier integrity, female reproductive tract abnormalities, altered mammary gland development, and reduced reproducibility, illustrating the importance of AHR function for normal development and physiology.^6^^,^^42^ AHR knockout rats generated by gene targeting methods were also insensitive to TCDD dependent induction of AHR-responsive genes and acute TCDD toxicity. However, they did not have the same vascular developmental abnormalities or hepatic defects as those observed in AHR knockout mice,^43^^,^^44^ suggesting that AHR has different roles in tissue development across rodent species.^43^

### B Aryl hydrocarbon receptor ligands

AHR ligands were traditionally characterized by their planar structure that allows them to interact with the PAS-B ligand binding domain of AHR.^42^^,^^45^ HAHs, such as TCDD, and other polychlorinated dibenzo-p-dioxins, polychlorinated dibenzofurans, and some dioxin-like polychlorinated biphenyls are among the most potent AHR agonists.^17^^,^^45^ Polychlorinated dibenzo-p-dioxins are a group of 75 related compounds referred to as congeners, whereas polychlorinated dibenzofurans and polychlorinated biphenyls consist of 135 and 209 related congeners, respectively. Of these chemicals, 7 polychlorinated dibenzo-p-dioxins, 10 polychlorinated dibenzofurans, and 12 dioxin-like polychlorinated biphenyls have TCDD such as toxicity via binding to the AHR.^46^ Because of their similar toxicological properties, a toxic equivalency (TEQ) approach is used to express the overall toxicity of complex mixtures of these chemicals as a single value. To calculate a TEQ, toxic equivalency factors (TEFs) for each individual congener are used. TEFs are determined by the relative toxicity of individual congeners to that of TCDD, which is considered the most toxic and set to 1.^47^ A TEQ is then calculated from the sum of the concentrations of individual congeners multiplied by their TEFs. The TEQ approach is commonly used to assess dioxin toxicity of human and environmental samples as well as food products.^48^, ^49^, ^50^ PAHs, such as B[*a*]P and 3-methylcholanthrene, bind with lower affinity to AHR. Over the last decades, a wide range of structurally diverse exogenous and endogenous compounds have been shown to either activate or inhibit AHR acting as agonists or antagonists, respectively.^45^^,^^51^ However, there are few publications that include ligand binding studies, which have led to some controversy in the field in terms of what constitutes a bona fide AHR ligand (reviewed in Sladekova et al^52^). In the simplest definition, a receptor is the target of a substance that is responsible for initiating a biological response, whereas a ligand binds to a receptor either orthosterically in the active site or allosterically at a distant and separate site, thereby indirectly enhancing or reducing the receptor’s ability to respond to the natural ligand.^53^ Ligand affinity is characterized by an affinity constant (K_d_), with low K_d_ values associated with high binding affinity. Longstanding methods used to determine ligand binding to AHR include a charcoal-dextran assay, a hydroxyapatite binding assay, and sucrose density centrifugation.^54^, ^55^, ^56^, ^57^ These assays have been used to quantitatively measure specific binding of radiolabelled ligands to AHR. In competitive binding assays, affinity is commonly expressed as a half maximal inhibitory concentration (IC_50_), which is determined by measuring the ability of a test ligand to compete for binding with a known ligand, such as radiolabelled TCDD or *β*-naphthoflavone.^58^^,^^59^ Ligands can act as agonists or antagonists depending on whether they activate or inhibit receptor responses. However, many AHR ligands have been identified from their ability to induce AHR-regulated reporter gene expression (ie, luciferase) in stable or transiently transfected reporter cell lines or through increases in the expression of AHR target genes. CYP1A1-driven reporter gene assays have been used by many to determine the effective concentration of a test compound to produce a half-maximal response (EC_50_).^60^^,^^61^ However, the outcomes observed in these complex cell systems might be due to indirect or alternative mechanisms. The use of drug affinity responsive target stability and cellular thermal shift assays (or simply thermal shift assay) will at least provide qualitative measures of a ligand-AHR interaction.^62^, ^63^, ^64^, ^65^, ^66^ Drug affinity responsive target stability are based on the premise that ligand binding often enhances the structural stability of proteins, protecting them from proteolytic digestion, whereas cellular thermal shift assays assume that ligand binding increases receptor stability under thermal denaturation. The establishment of a microscale thermophoresis-based approach using purified AHR and ARNT offers a promising higher throughput approach to quantitatively determine ligand binding to AHR, including the determination of K_d_ values.^67^^,^^68^

AHR modulators come from both exogenous and endogenous sources. Exogenous modulators include environmental pollutants, synthetic chemicals, pharmaceuticals, and numerous dietary compounds. Endogenous modulators are generated through cellular metabolism with many being derived from the metabolic breakdown of tryptophan or by microbiota communities in the gut, oral cavity, respiratory tract, and skin.^69^ These ligands or modulators work as direct agonists or antagonists, proligands that are metabolically converted to their active forms, or activators/inhibitors that indirectly affect AHR in a context-dependent manner.^52^ These diverse actions of AHR ligands have led to the concept of selective AHR modulators (SAHRMs), which considers the tissue/cell-specific AHR agonist and antagonist activities.^70^ In addition to tissue- and cell-specificity, activation of AHR by SAHRMs can induce the transcription of selective genes that could potentially favor antitumorigenicity while avoiding the transcription of oncogenes. Despite promising preclinical results, the development of SAHRMs has not yet proven efficient in the clinical setting, in part because of the difficulties in predicting the effect and response to such compounds.^70^ Moreover, many AHR ligands also activate or inhibit other transcription factors, such as ER*α* (ESR1; NR3A1), ER*β* (ESR2; NR3A2), and pregnane X receptor (NR1I2).^71^^,^^72^ AHR ligands also vary in their intrinsic efficacy, leading to tissue- and target gene-specific differences in responsiveness.^73^ These differential intrinsic ligand efficacies and AHR promiscuity combined with ligand crosstalk might explain many of the context-dependent responses attributed to different AHR ligands.

#### 1 Exogenous ligands

**Fig. 2:**
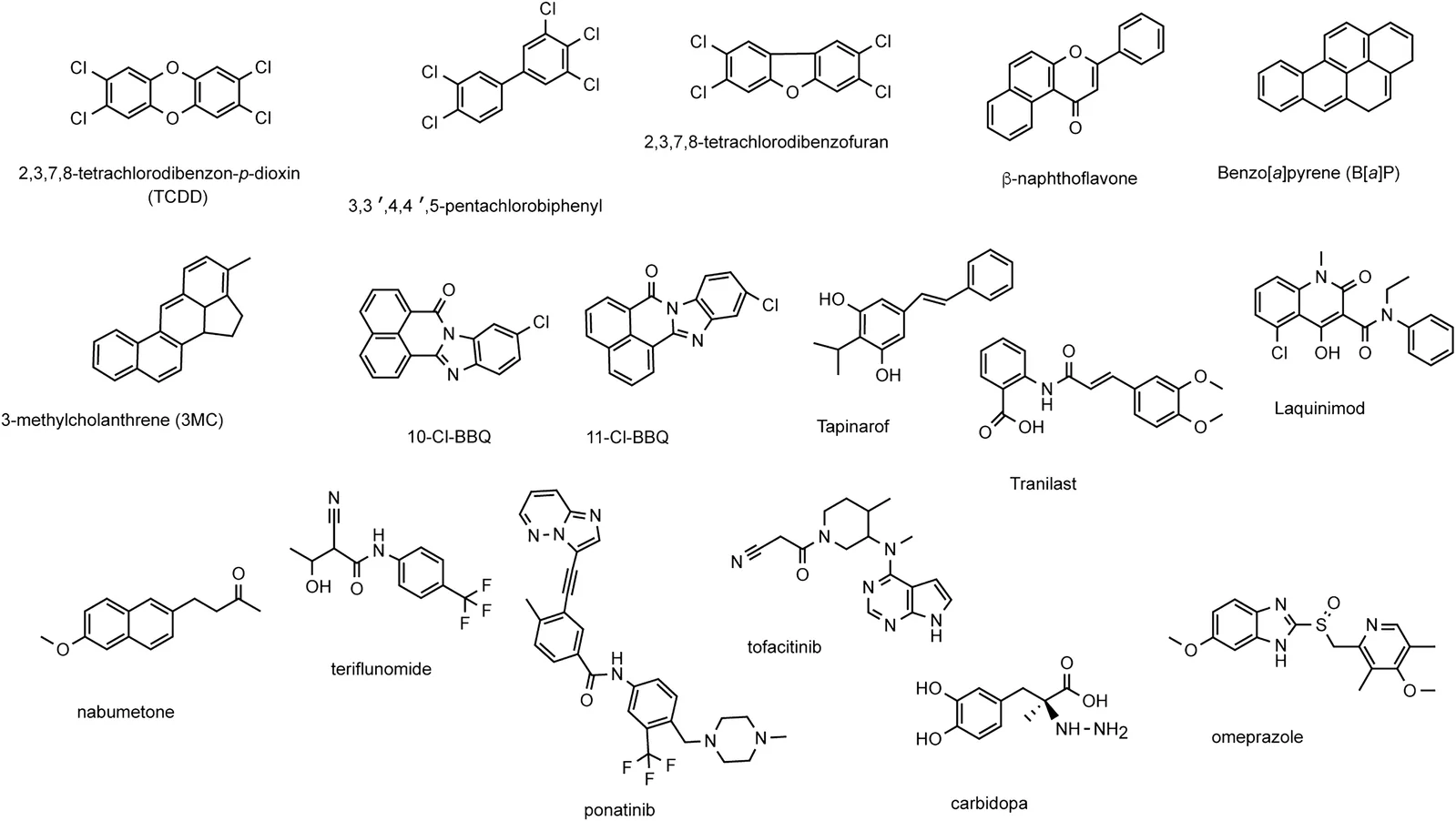
Chemical structures of some exogenous AHR activating chemicals.

HAHs, such as TCDD (K_d_: 0.5 nM) 2,3,7,8-tetrachlorodibenzofuran and 3,3′,4,4′,5-pentachlorobiphenyl (K_d_: 1.3 nM) are environmental contaminants that exhibit high affinity and prolonged binding to AHR, causing sustained AHR activation (Fig. 2).^74^ This increased and stable AHR response has been suggested to contribute to their toxicity, although recent findings suggest that this may not be the case for AHR regulated immune responses.^75^ PAHs, such as B[*a*]P, are products of combustion and industrial processes that are found in tobacco smoke, air pollution and charbroiled food. PAHs generally have a lower affinity for AHR than HAHs. 3-Methylcholanthrene is a synthetic PAH, whereas *β*-naphthoflavone is a synthetic flavone and is routinely used as a nontoxic AHR agonist.

Tapinarof (3,5-dihydroxy-4-isopropylstilbene) is the first-in-class nonsteroidal, topical AHR agonist (K_d_: 100–200 nM) that was approved by the US Food and Drug Administration (FDA) in 2022 for the treatment of plaque psoriasis and in 2024 for the treatment of atopic dermatitis.^76^, ^77^, ^78^ The clinical approval of tapinarof was a milestone in the efforts to establish AHR as a viable therapeutic target. Tapinarof leverages AHR’s anti-inflammatory and antioxidant actions to reduce skin inflammation.^79^ Laquinimod is a quinoline-3-carboxamide and AHR agonist developed by Active Biotech and Teva that was investigated as an oral treatment for multiple sclerosis and Huntington disease.^80^^,^^81^ There is also interest in pursuing laquinimod for the treatment of inflammatory bowel disease.^82^ 10-Chloro-7H-benzimidazo[2,1-a]benzo[de]iso-quinolin-7-one and its analog 11-chloro-7H-benzimidazo[2,1-a]benzo[de]iso-quinolin-7-one are rapidly metabolized AHR activators and immune modulators discovered from small molecule library screening.^83^ Drug repurposing screening strategies have identified other FDA approved agents that have AHR modulating activities. Such screening efforts have identified the cyclooxygenase inhibitor, nabumetone, and the dihydroorotate dehydrogenase inhibitor, teriflunomide, as AHR activators.^84^ The tyrosine kinase inhibitor, ponatinib, and the Janus kinase (JAK) inhibitor, tofacitinib, were also reported to reduce TCDD-dependent increases in CYP1A1 levels.^85^ The proton-pump inhibitor omeprazole has been identified as an indirect AHR activator, proagonist and proantagonist depending on the metabolite generated and in a species-specific manner.^52^ Carbidopa, used to treat Parkinson disease, was reported to activate AHR.^86^ Tranilast is an AHR activator and clinically approved antiallergic agent used in Japan, South Korea, and China, but it is not approved by the FDA.^87^

#### 2 Exogenous synthetic aryl hydrocarbon receptor antagonists

**Fig. 3:**
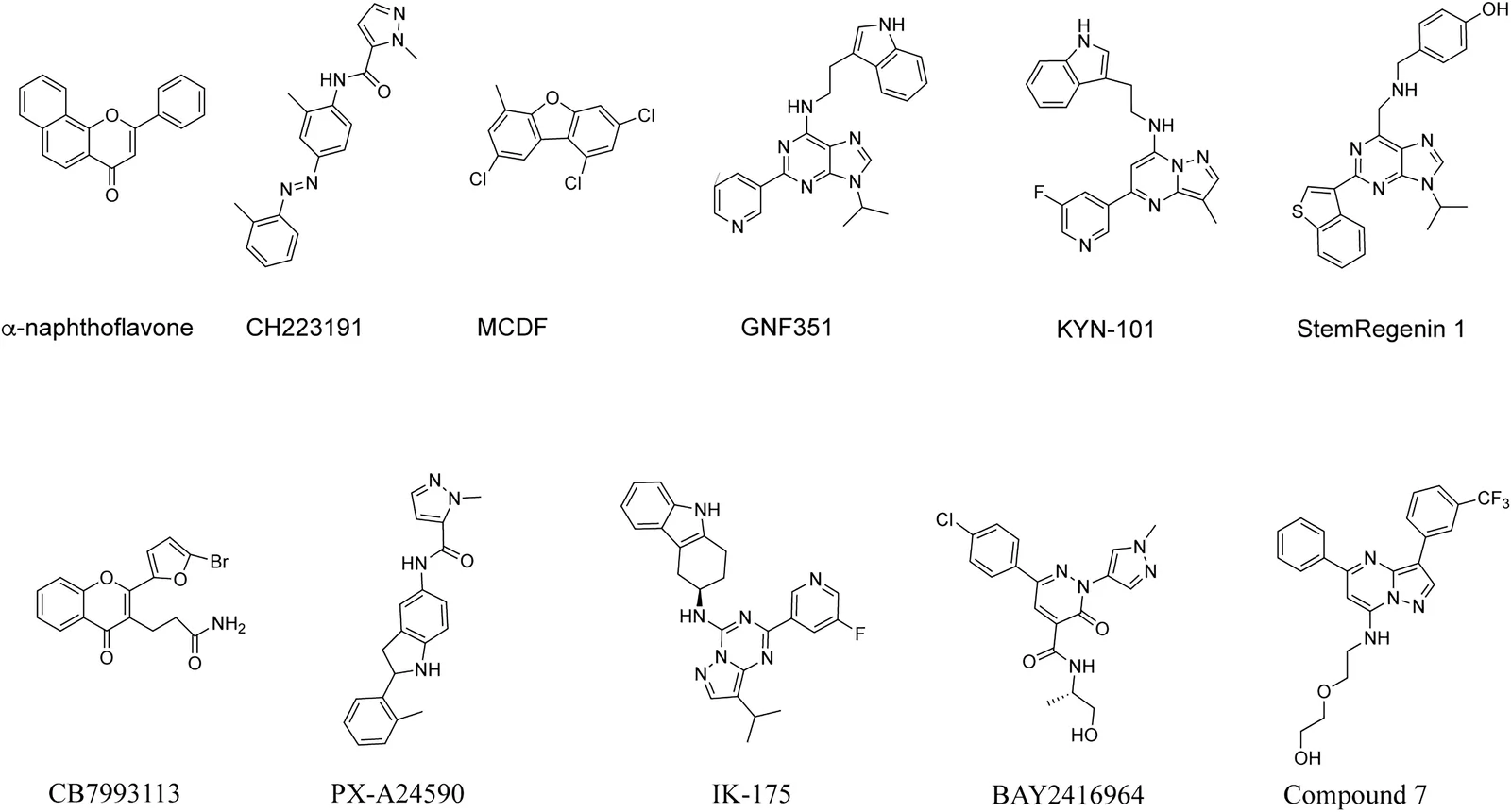
Chemical structures of some exogenous AHR antagonists.

In addition to the multitude of chemicals that activate AHR, several AHR antagonists have been identified and characterized (Fig. 3).^88^ Because AHR is a key regulator of tumor progression, exhibiting both tumor cell intrinsic properties and antitumor immunity effects, inhibiting its activity has emerged as an exciting new cancer therapy. *α*-Naphthoflavone (IC_50_: 0.06 *μ*M) and 6-methyl-1,3,8-trichlorodibenzofuran (IC_50_: 40 nM) are 2 of the first reported AHR antagonists, with the former also exhibiting partial agonist activity at higher doses.^89^, ^90^, ^91^, ^92^ *α*-Naphthoflavone is also a potent competitive inhibitor of several cytochrome P450 enzymes, complicating its use as an AHR antagonist.^93^ 6-Methyl-1,3,8-trichlorodibenzofuran is classified as a SAHRM because it acts as an antiestrogen in cancer cells, potentially inhibiting tumor growth, and can antagonize the toxic effects of TCDD in a context-dependent manner.^94^ A chemical library screen identified CH223191 (2-methyl-2H-pyrazole-3-carboxylic acid (2-methyl-4-o-tolylazo-phenyl)-amide) as a novel compound that exhibits ligand-selective AHR antagonism.^95^^,^^96^ CH223191 (IC_50_: 0.03 *μ*M) inhibited the ability of TCDD and related halogenated hydrocarbons to activate AHR but did not antagonise the AHR activating activity of certain PAHs, flavonoids, or indirubin, suggesting that it acts as a selective AHR antagonist.^97^ Various resveratrol derivatives are selective AHR antagonists.^98^ The FDA-approved antileprosy agent, clofazimine, inhibits B[*a*]P, and TCDD dependent AHR activation.^99^ StemRegenin 1, a purine derivative, was identified from a library screen of 100,000 heterocycles to identify compounds that promote hematopoietic stem cell. Mechanistically, StemRegenin 1 stimulates hematopoietic stem cell expansion by acting as an antagonist of AHR (IC_50_: 40 nM).^100^^,^^101^ Other computational screens identified antagonists GNF351 (K_d_: 62 nM, IC_50_: 8.5 nM) and CB7993113 (IC_50_: 330 nM).^102^, ^103^, ^104^, ^105^ GNF351 was found to be poorly absorbed and extensively metabolized after oral administration, which limits its ability to inhibit AHR activation in vivo.^106^ The indole derivative AHR antagonist, PX-A24590 (5, IC_50_: 60 nM), was developed by Phenex Pharmaceuticals AG.^107^ Structure based drug design approaches were used to identify a pyrazolo[1,5-*a*]pyrimidine-based AHR antagonist, termed compound 7 (IC_50_: 31 nM).^108^ The AHR antagonists kynurenine-101 (IC_50_: 22 nM), IK-175 (IC_50_: 31 nM), and BAY2416964 (IC_50_: 22 nM) have potential as anticancer therapeutics.^88^^,^^109^^,^^110^ Similar to CH223191, many of the abovementioned antagonists are selective AHR inhibitors rather than pure antagonists.^108^ For example, BAY2416964, GNF351, and compound 7 prevent TCDD, but not indirubin- nor tapinarof-induced *Cyp1a1* reporter gene activity.^108^ Currently, IK-175, developed by Ikena Oncology, and BAY2416964, developed by Bayer, are being investigated in combination with immune checkpoint therapy against advanced solid tumors in phase 1 clinical trials (NCT04200963 and NCT04999202, respectively).^111^^,^^112^

#### 3 Dietary ligands

**Fig. 4:**
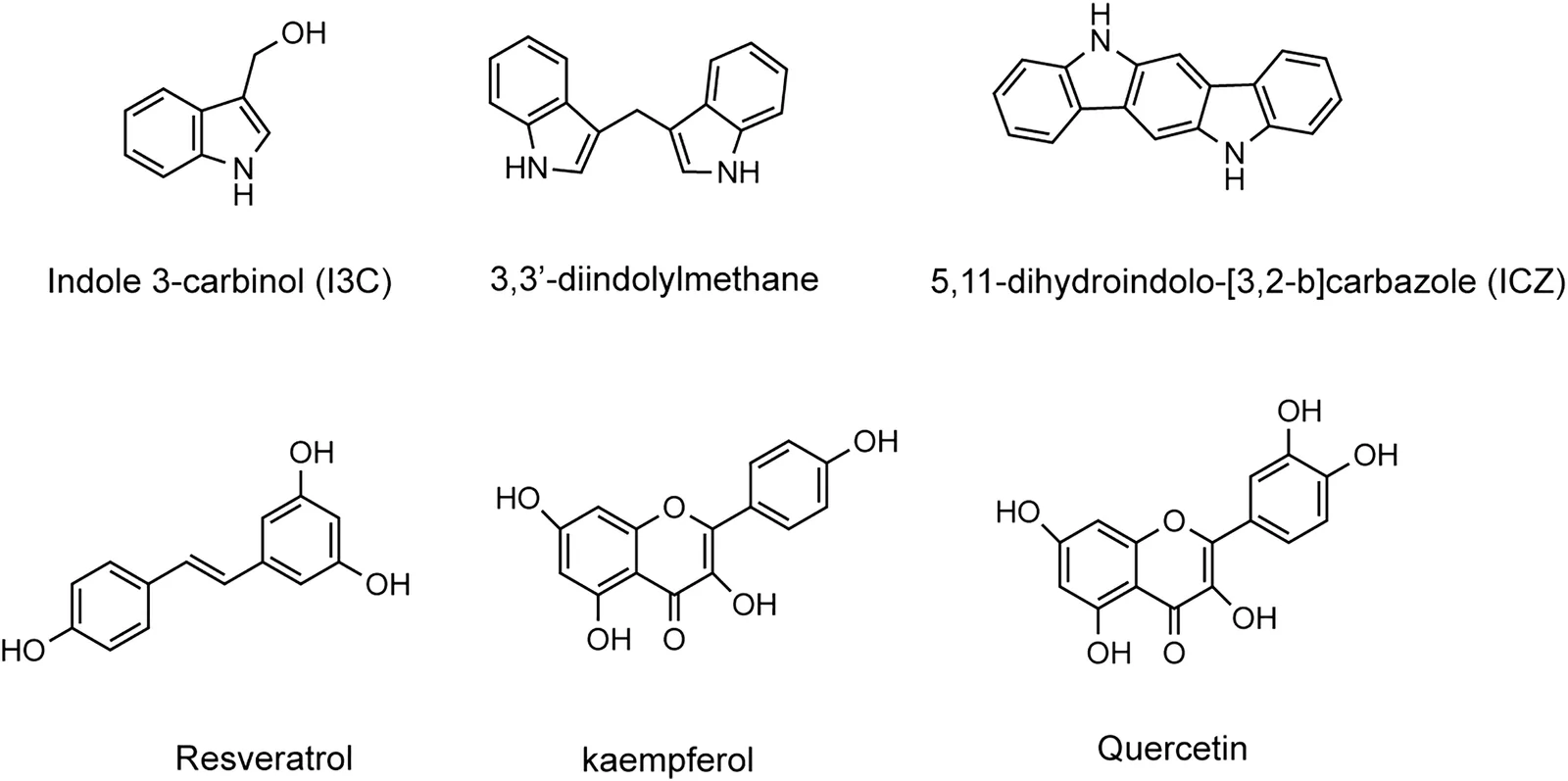
Chemical structures of select dietary AHR modulating chemicals.

There are several different dietary polyphenols and phytochemical compounds that modulate AHR, including indoles, flavonoids, stilbenes, and carotenoids (Fig. 4).^69^ Indole 3-carbinol (I3C) was the first described dietary modulator of AHR and is an example of a proligand of AHR. Cruciferous vegetables such as broccoli, cabbage, brussels sprouts, and kale contain considerable amounts of glucobrassicin. During chewing, plant-based myrosinases enzymatically break down glucobrassicin, leading to the formation of I3C, which undergoes acid-catalyzed condensation to produce 3,3′-diindolylmethane (DIM), trace amounts of 5,11-dihydroindolo-[3,2-*b*]carbazole and a cyclic triindole.^113^ DIM (IC_50_: 5 *μ*M) is a weak or partial AHR agonist, whereas 5,11-dihydroindolo-[3,2-*b*]carbazole (K_d_: 190 nM) is a high affinity AHR ligand.^114^, ^115^, ^116^ DIM activates other nuclear receptors, including NR3A1, NR3A2, NR1I2, and constitutive androstane receptor (NR1I3).^117^, ^118^, ^119^

Flavonoids are the most abundant class of dietary phenolic chemicals, with more than 8000 different chemicals divided into different chemical subclasses.^120^ They are extensively used in the pharmaceutical industry, partly because of their antioxidant, anti-inflammatory and anticancer effects as well as their ability to improve immune response to infection.^121^ AHR modulators are primarily found among the flavones, flavonols, flavanones, and isoflavones and exhibit both agonist and antagonist effects in a context dependent manner (reviewed by Pinto et al^122^ and Goya-Jorge et al^123^). Quercetin and kaempferol were among the first flavonoids to be identified as AHR modulators, with quercetin activating and kaempferol inhibiting AHR signaling in treated MCF7 human breast cancer cells.^124^ Other studies reported that quercetin acts as an antagonist, whereas kaempferol was reported to be a weak agonist,^125^ showing that the actions of AHR modulating flavonoids are context dependent. Resveratrol (IC_50_: 6 *μ*M) present in nuts, berries, and grapes has been reported to act as an AHR antagonist by reducing TCDD-dependent induction of CYP1A1 and recruitment of AHR to *CYP1A1* promoter.^126^, ^127^, ^128^, ^129^, ^130^

However, resveratrol and some flavonoids are proposed to be indirect AHR activators, because they inhibit CYP1 enzyme activity, which reduces the degradation of other endogenous AHR activators.^131^ Although several in vitro studies support the ability of many structurally diverse chemicals to bind to the AHR (reviewed in Denison and Faber^71^), proposed AHR agonists should be carefully considered in the absence of binding studies.

#### 4 Endogenous ligands

**Fig. 5:**
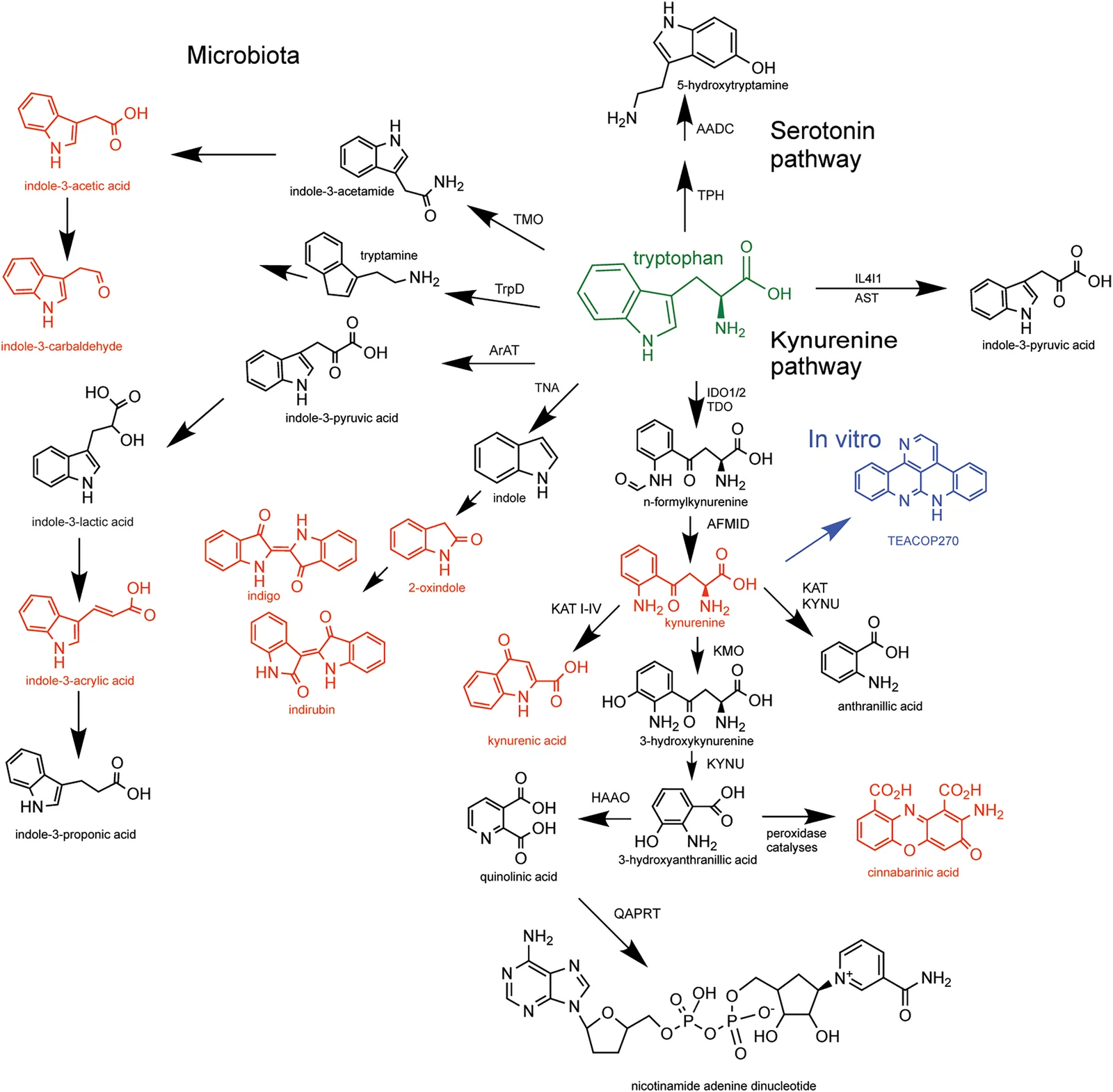
Pathways of tryptophan metabolism via kynurenine, indoles and serotonin. Chemical structures shown in red indicate AHR modulating chemicals. Trace extended aromatic condensation product 270 (TEACOP270) is shown in blue to indicate that is formed in vitro.

Many endogenous AHR ligands are generated by differential metabolic breakdown of the essential amino acid tryptophan.^132^ UV light derived tryptophan photoproduct 6-formylindolo[3,2-b]carbazole (FICZ) is a potent AHR agonist that is generated in the skin with a higher affinity for human AHR (K_d_: 0.07 nM) compared with TCDD (K_d_: 0.5 nM).^133^^,^^134^ FICZ is produced in cell culture media from tryptophan and contributes to positive AHR responses depending on assay conditions.^135^ After absorption of tryptophan, approximately 95% of tryptophan is metabolized into n-formylkynurenine by the rate-limiting enzymes indoleamine-2,3-dioxygenase (IDO1), IDO2 or tryptophan-2,3-dioxygenase (TDO2) (Fig. 5). Increased activity of these enzymes depletes cellular tryptophan while increasing intermediate metabolites, which negatively affects cellular function and survival.^136^ N-formylkynurenine is converted to the AHR modulator, kynurenine, by arylformamidase.^137^ It was recently reported that prolonged storage of kynurenine results in the generation of at least 2 trace extended aromatic condensation products with high affinity for AHR in vitro. Trace extended aromatic condensation product 270 has a reported EC_50_ of 0.3 nM in CYP1A1 reporter gene assays.^138^ These findings support that kynurenine acts as a proagonist rather than a direct AHR ligand. Kynurenine aminotransferases (I–IV) catalyze the conversion of kynurenine to AHR activator kynurenic acid. The L-amino acid oxidase, interleukin-4-induced-1 (IL4I1), has also been shown to metabolize tryptophan to indole-3-pyruvic acid, which is subsequently converted to AHR agonists indole-3-aldehyde and kynurenic acid.^139^ Kynurenine can also be converted to anthranilic acid by kynureninase or to 3-hydroxykynurenine by kynurenine 3-monooxygenase and then to the AHR activator, xanthurenic acid by kynurenine aminotransferases. 3-Hydroxykynurenine is metabolized to 3-hydroxyanthranilic acid and further converted to quinolinic acid by 3-hydroxyanthranilate-3,4-dioxygenase. 3-hydroxyanthranilic acid is converted to the AHR ligand cinnabarinic acid,^140^ which has recently been shown to protect against metabolic dysfunction associated with steatohepatitis.^141^ Quinolinic acid phosphoribosyltransferase and NAD synthase catalyze the metabolization of quinolinic acid to nicotinamide adenine dinucleotide (NAD^+^).

A small amount of tryptophan is converted to 5-hydroxytryptophan by tryptophan hydroxylase 1 in the brain and by tryptophan hydroxylase 2 in the gut before it is decarboxylated to 5-hydroxytryptamine (serotonin) by aromatic L-amino acid decarboxylase. More than 90% of the serotonin production in the body occurs in the gut by enterochromaffin cells. Serotonin has been reported to be an indirect AHR modulator through its effects on CYP1A1 activity.^142^ Gut microbiota bacteria metabolize tryptophan into several different products, including indole by tryptophanase, indole-3-pyruvate by aromatic amino acid aminotransferase, tryptamine catalyzed by tryptophan decarboxylase and indole-3-acetamide by tryptophan 2-monooxygenase (Fig. 5). Indole, indole-3-pyruvate and indole-3-acetamide are further metabolized to 2-oxindole, 2,8-dihydroxyquinoline 3-methylindole, indole-3-lactic acid, indole-3-acrylate, indole-3-acetic acid, indole-3-ethanol, indole-3-aldehyde, indole-3-acetaldehyde, and indole-3-propionic acid.^143^, ^144^, ^145^ Although multiple microbial generated indoles have been reported to be weak AHR ligands, only indole, 2-oxindole and indole-3-acetic acid, kynurenic acid, and 2,8-dihydroxyquinoline induced *Cyp1a1* reporter gene activity at concentrations detected in human feces.^144^^,^^146^ Interestingly, indole binds human AHR with higher affinity compared with mouse AHR, which is thought to occur through an evolutionary adaptation by the AHR.^147^ Indirubin and indigo are bisindole alkaloids and naturally occurring pigments that are also highly potent endogenous activators of AHR found in human urine. Indirubin and indigo are generated from tryptophan via indole by gut microbiota and are also the bioactive components found in indigo naturalis that is used in traditional Chinese medicine for their anti-inflammatory, antibacterial, antiviral, and immunomodulatory activities.^148^ Ellagitannins found in nuts and fruits are metabolized by microbiota to urolithin A and B, which act as AHR antagonists.^149^

Other potential endogenous AHR modulators include 2-(1′H-indole-3′-carbonyl)-thiazole-4-carboxylic acid methyl ester (ITE), which was isolated from porcine lungs with a high reported affinity for AHR (EC_50_: 5 nM).^150^^,^^151^ However, ITE has not been directly identified in humans, and it has been reported to be produced by different bacterial strains found in compost, raising doubt as to whether ITE is an endogenous AHR ligand.^52^ Several other endogenous compounds have been described to act as AHR modulators,^130^ including lipoxin A4 and several prostaglandins that weakly activate AHR,^152^^,^^153^ and heme-metabolites bilirubin and biliverdin.^154^^,^^155^

### C Aryl hydrocarbon receptor mechanism of action

#### 1 Canonical aryl hydrocarbon receptor signaling

**Fig. 6:**
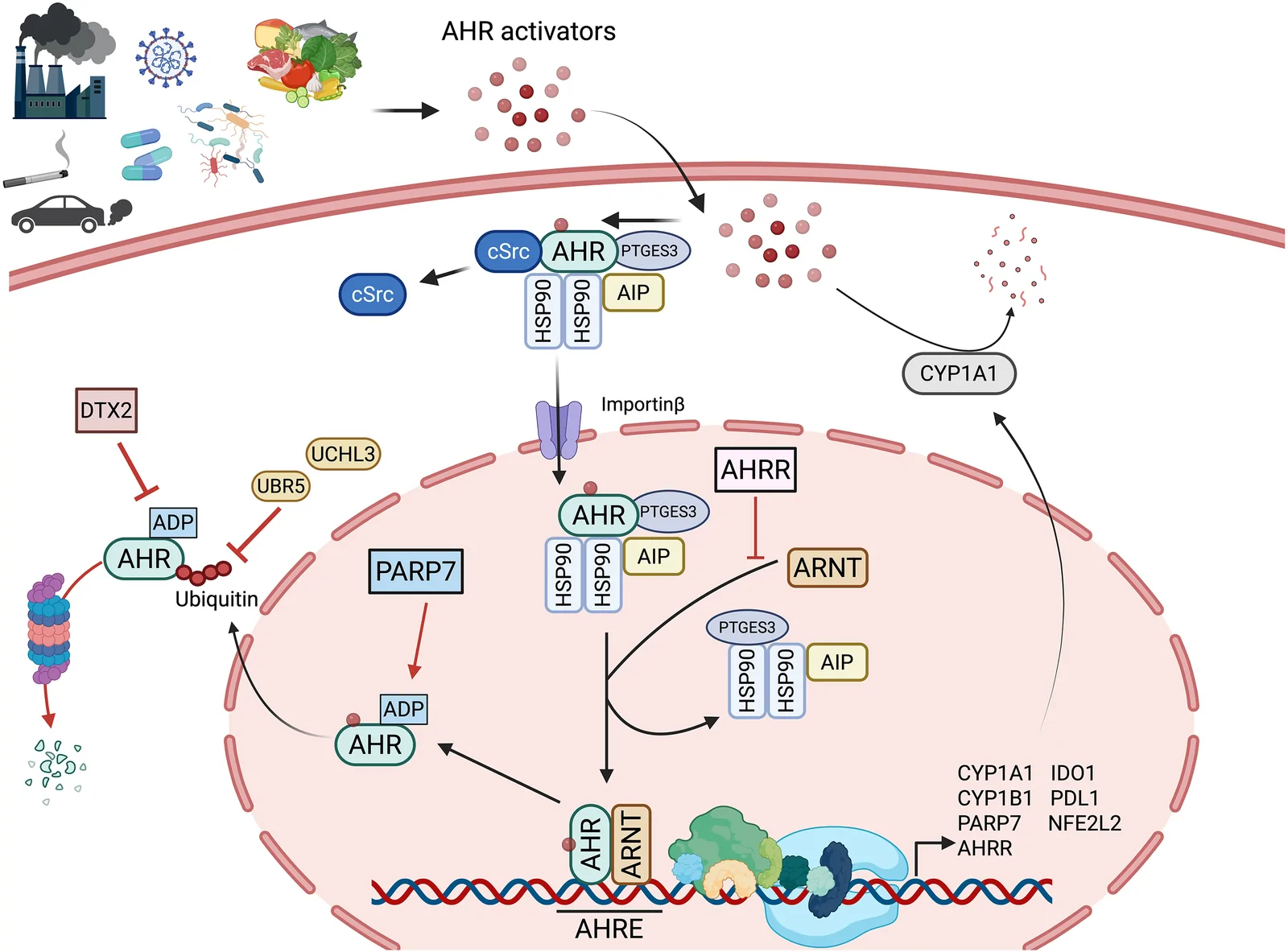
Canonical AHR signaling pathway. Inactive AHR is in the cytosol bound to a HSP90 dimer, XAP2, PTGES3, and soluble tyrosine kinase (cSrc) forming a cytosolic complex. AHR activators diffuse through the cell membrane and bind AHR facilitating nuclear translocation mediated by importin-*β*. In the nucleus, AHR heterodimerizes with ARNT and dissociates from the complex proteins. The AHR-ARNT heterodimer binds AHREs leading to the recruitment of coregulators and increasing the transcription of AHR target genes. AHR is negatively regulated (marked by red arrow/inhibition sign) by competitive binding of AHRR with ARNT, enzymatic degradation of AHR agonists by CYP enzymes, mono-ADP-ribosylation by PARP7, ubiquitination and proteolytic degradation. AHR protein stability is modulated by the actions of deltex E3 ubiquitin ligase 2 (DTX2), which removes PARP7-dependent ADP-ribose from AHR, and by ubiquitin protein ligase E3 component N-recognin 5 (UBR5) and ubiquitin carboxyl-terminal hydrolase L3 (UCHL3), which prevent ubiquitination of AHR. Created in BioRender. Matthews, J. (2026) https://BioRender.com/gus0n14.

To fully appreciate AHR’s pharmacological potential, it is essential to understand the complex cellular signaling pathways regulated by AHR as well as how AHR levels are regulated (Fig. 6). In what is termed the canonical AHR signaling, inactive AHR resides in the cytosol in a multi-protein complex consisting of heat shock protein 90 (HSP90), AHR interacting HSP90 cochaperone (AIP, also known as hepatitis B virus X-associated protein 2 (XAP2) and aryl hydrocarbon receptor-associated protein 9), PTGES3 (also known as p23), which is a molecular cochaperone and soluble tyrosine kinase.^156^^,^^157^ This multiprotein complex stabilizes a ligand receptive conformation and inhibits ligand-independent nuclear translocation of AHR. Upon ligand binding, AHR undergoes a conformational change, exposing the nuclear localization signal, resulting in the importin-*β*-dependent nuclear translocation of the complex. Once in the nucleus, AHR dissociates from the multiprotein complex and heterodimerizes with ARNT. The AHR-ARNT heterodimer binds to AHR response elements (AHREs, also known as xenobiotic response elements or dioxin response elements), which are defined by the consensus motif, 5′-TNGCGTG-3′. The DNA-bound AHR-ARNT complex recruits coregulator proteins and histone modifying enzymes to regulate the expression of its target genes, including *CYP1A1*, *CYP1A2*, *CYP1B1*, *AHRR*, and *PARP7* (also known as TCDD-inducible poly-ADP ribose polymerase [TIPARP]) (Fig. 6).^17^^,^^158^ Activation of AHR triggers its ubiquitination and degradation by the 26S proteasome in a process involving the ubiquitin ligase cullin-4B (Cul4B).^159^^,^^160^ Interestingly, the E3 ubiquitin ligase, ubiquitin protein ligase E3 component N-recognin 5 and the deubiquitylase, ubiquitin carboxyl terminal hydrolase L3, have been independently shown to stabilize AHR, highlighting the complexity of AHR protein regulation.^161^^,^^162^

#### 2 Feedback regulation of aryl hydrocarbon receptor signaling

AHR signaling is modulated by 3 different negative feedback mechanisms: ligand metabolism, AHRR-mediated repression, and PARP7-dependent inhibition and degradation. The AHR-dependent induction of CYP1A1, CYP1A2, and CYP1B1 enzymes results in the metabolism and subsequent secretion of many AHR agonists in a process known as adaptive metabolism.^163^ For example, activation of AHR by PAHs leads to the upregulation of CYP1 monooxygenase enzymes and their excretion, representing a classic feedback loop in which the substrate induces its own metabolism.^42^^,^^164^ High levels of PAHs can lead to an increase in metabolically reactive and toxic intermediates via the AHR-CYP1A1 axis.^165^^,^^166^ There was a longstanding notion that AHR ligands were not pursued in pharmaceutical development because of the induction of CYP1A1 as well as their potential TCDD-like properties. However, CYP1A1 induction and/or AHR activation was not synonymous with dioxin-like toxicity.^167^ Together with the identification of new AHR ligands with potential therapeutic applications has led to a reconsideration of the strategy.^168^ Interestingly, CYP1A1 overexpressing mice mimicked the sensitivity of *Ahr*^*−/−*^ mice to intestinal inflammation because of enhanced metabolism of endogenous AHR ligands.^169^ These studies highlight the importance of the AHR-CYP1A1 axis in normal physiology and inflammation.

AHRR was reported to negatively regulate AHR by competitively binding to ARNT.^170^ However, this mechanism has come under scrutiny since overexpression of ARNT or mutation of the DNA binding domain of AHRR failed to rescue AHR activity.^171^^,^^172^ This led to a proposed mechanism of transrepression where AHRR competes with AHR for co-activator proteins.^171^ *Ahrr*^*−/−*^ mice exhibited higher levels of *Cyp1a1* mRNA induction in the skin, stomach, and spleen but not in the liver, lung, heart, or other tissues, suggesting that AHRR regulation of AHR occurs in a tissue specific manner. They also had a delayed response to B[*a*]P-induced skin carcinogenesis, suggesting that increased CYP1A1 levels favor detoxification rather than metabolic activation of carcinogens like B[*a*]P.^173^ However, the sensitivity of *Ahrr*^*−/−*^ mice to TCDD toxicity has not been determined.

PARP7 is an AHR target gene and a member of the ADP-ribosyltransferase (ART) family (also known as the PARP family). ART family of proteins transfer a single (mono-ART) or several (poly-ART) ADP-ribose molecules onto themselves or target proteins using NAD^+^ as substrate.^174^ Although the official gene name for *PARP7* is *TIPARP*, in support of updated nomenclature for mammalian ARTs it will be referred to as *PARP7* hereafter.^174^ Loss of PARP7 or treatment with the PARP7 inhibitor, RBN2397, enhanced AHR signaling.^172^ *Parp7* null and *Parp7* catalytic mutant mice are highly sensitive to the TCDD-induced hepatotoxicity and lethality.^175^^,^^176^ PARP7 was found to negatively regulate AHR by mono-ADP-ribosylation that promotes the proteolytic degradation of AHR.^175^, ^176^, ^177^ NAD^+^, an important coenzyme in energy metabolism, is used as a substrate by PARP7 to inhibit AHR signaling, which provides a further link between tryptophan metabolism and AHR regulation. Emerging studies describing reciprocal crosstalk between ADP-ribosylation and ubiquitination provide a link to the proteasomal degradation of ADP-ribosylated proteins.^178^^,^^179^ The E3 ligase, deltex E3 ubiquitin ligase 2, was also recently found to degrade ADP-ribosylated PARP7 and AHR, leading to rapid shutdown of AHR-mediated transcription.^180^^,^^181^ The CUL4B E3 ligase complex and PARP7 were reported to work in concert to regulate the proteasomal degradation of AHR,^182^ whereas SUMOylation stabilized AHR by inhibiting its ubiquitination.^183^

#### 3 Noncanonical genomic aryl hydrocarbon receptor signaling

**Fig. 7:**
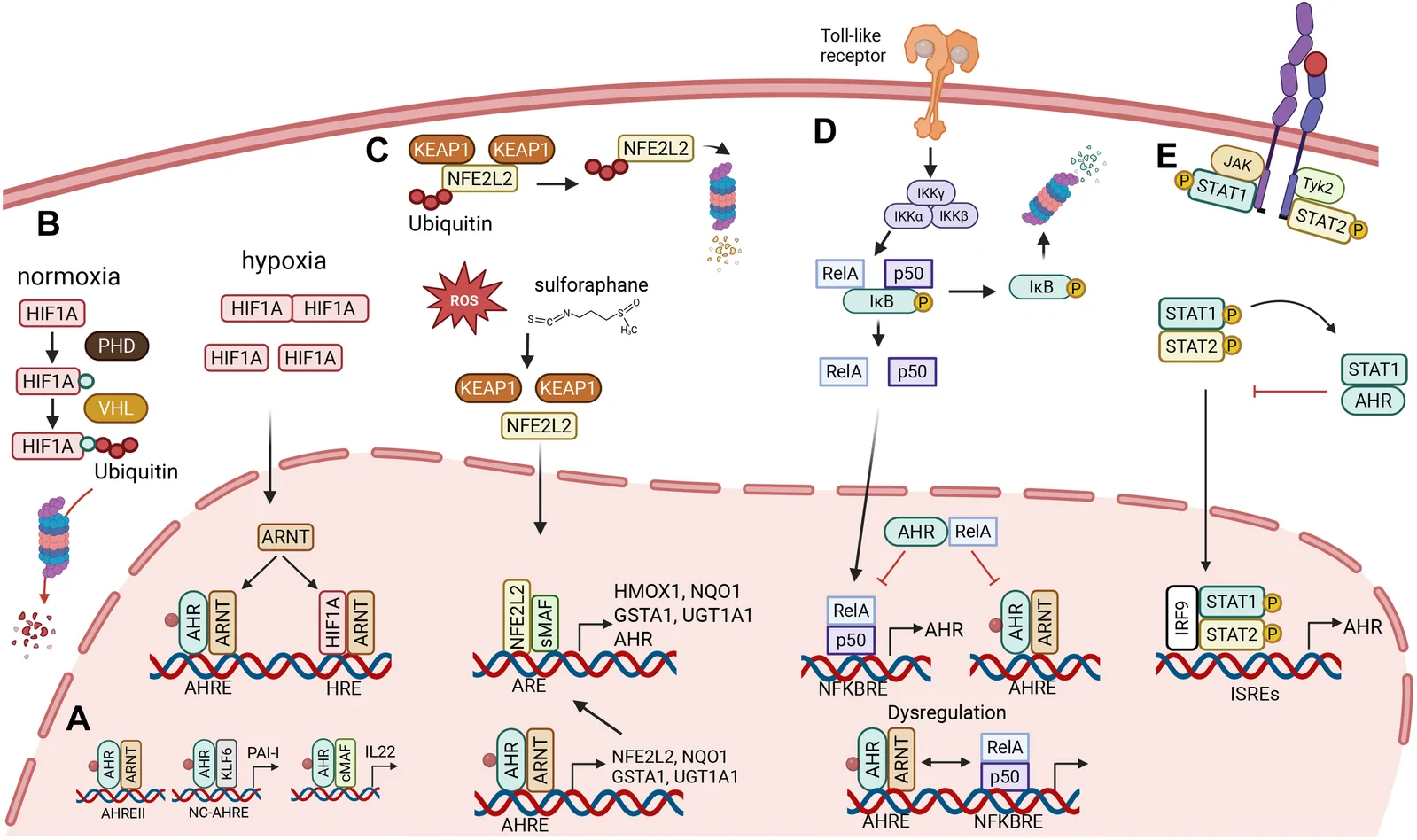
Examples of noncanonical AHR signaling. (A) AHR regulates some genes through binding to AHREII and via nonconsensus AHRE (NC-AHRE) sequences or by interacting with other transcription factors. (B) Reciprocal regulation of AHR and HIF-1A by competition for ARNT under stimulation of AHR ligands or hypoxia, respectively. (C) Reciprocal regulation of AHR and NFE2L2 under stimulation of AHR ligands or reactive oxygen species (ROS), respectively. (D) AHR and NFκB binds to the same promotor region of target genes causing dysregulated transcription. (E) Activation of STAT1, STAT2, interferon regulatory factor 9 (IRF9) induce expression of AHR, whereas AHR binds STAT1 and thereby inhibits its phosphorylation and activation. ARE, antioxidant response elements; HRE, hypoxia response elements; ISRE, IFN-stimulated response elements; PHD, prolyl hydroxylase domain; Ub, ubiquitin; VHL, von Hippel-Lindau protein. Created in BioRender. Matthews, J. (2026) https://BioRender.com/t76hmx9.

AHR signals through several noncanonical genomic pathways to alter the activity or crosstalk with other signaling pathways (Fig. 7).^184^ The AHR-ARNT heterodimer modulates gene expression through tethering to an unknown transcription factor, which binds to AHRE-II element with the consensus sequence CATG(N6)C[T/A]TG.^185^ AHRE-II elements have been identified in 36 genes across mouse, rat and human genomes.^186^ AHR interacts with Kruppel-like factor 6 to regulate target gene expression through nonconsensus AHRE sequence independently of ARNT.^187^ Kruppel-like factor 6 is a zinc finger transcription factor and tumor suppressor that regulates cell proliferation, differentiation, apoptosis and hepatocyte glucose, and lipid homeostasis.^188^ The serine protease inhibitor, plasminogen activator inhibitor 1 and cyclin-dependent kinase inhibitor 1A (also known as p21Cip1), a cyclin-dependent kinase inhibitor of the G1-phase of the cell cycle, are regulated by AHR-Kruppel-like factor 6 complex binding to nonconsensus AHRE elements (Fig. 7A).^189^^,^^190^ AHR also interacts with cellular musculoaponeurotic fibrosarcoma to regulate the expression of *interleukin 10* (*IL10*) and *IL21* (Fig. 7A).^191^

Competitive heterodimerization with ARNT (HIF1B) occurs when both AHR and HIF-1A pathways are activated (Fig. 7B). Dual activation of these pathways creates a reciprocal and opposing signaling crosstalk. HIF1A signaling dominates over AHR signaling under hypoxic conditions, whereas HIF1A response is reduced after AHR agonist-treatment.^192^ In models of cystic fibrosis, HIF1A induction reduced AHR responses leading to hypoxia-driven inflammation, suggesting that AHR might restore immune homeostasis, and improve lung function.^193^ In cancer, both HIF1A and AHR inhibitors are being developed for the treatment of solid tumors, but inhibition of one pathway could promote the protumorigenic effects of the other. Recently, it was suggested that combination therapy targeting both HIF1A and AHR might provide greater efficacy than targeting each one alone.^194^

Nuclear factor erythroid-derived 2-like 2 (NFE2L2; also known as nuclear factor erythroid 2-related factor 2) is a master regulator of the cellular response to oxidative stress and an AHR target gene. NFE2L2 is a member of the Cap‘n’Collar protein family.^195^^,^^196^ Under normal conditions, NFE2L2 is in the cytoplasm bound to the redox-regulated substrate adaptor cullin 3-containing E3 ubiquitin ligase, kelch-like ECH-associated protein 1 (KEAP1). KEAP1 is a cysteine rich protein that targets NFE2L2 for ubiquitylation and subsequent proteasomal degradation.^197^ In response to oxidative stress or dietary compounds, such as sulforaphane (present in cruciferous vegetables), oxidation of critical cysteine residues causes a conformational change in KEAP1, resulting in the release and subsequent nuclear translocation of NFE2L2. Nuclear NFE2L2 forms a heterodimer with small musculoaponeurotic fibrosarcoma protein and the heterodimer binds to antioxidant response elements in the regulatory regions of its target genes, such as *heme oxygenase-1* (*HMOX-1*), *NAD(P)H quinone oxidoreductase 1* (*NQO1*), *glutathione S-transferase A1* (*GSTA1*), and *uridine diphosphate glucuronosyltransferase 1A1* (*UGT1A1*).^198^ AHR-NFE2L2 crosstalk occurs at multiple levels, including via direct reciprocal positive regulation in which AHR regulates NFE2L2 levels and NFE2L2 regulates AHR levels (Fig. 7C). HMOX-1 catalyzes the degradation of heme into carbon monoxide, iron (II), and AHR ligands, biliverdin and bilirubin.^199^ AHR-mediated induction of cytochrome P450 enzymes increases oxidative stress stimulated NFE2L2 signaling.^200^, ^201^, ^202^, ^203^ AHR and NFE2L2 share several target genes, including *NQO1*, *GSTA1*, and *UGT1A1*. The signaling crosstalk between AHR and NFE2L2 influences barrier organ function, contributing to the therapeutic benefit and anti-inflammatory actions of tapinarof in skin pathologies.^204^

AHR regulates inflammatory response through crosstalk with Toll-like receptors and members of the nuclear factor *κ*B (NF*κ*B) family (Fig. 7D). NF*κ*B transcription factors are involved in essential pathways regulating immune responses and inflammation, but also cell survival, cell differentiation, and cancer.^205^, ^206^, ^207^ NF*κ*B exists as a family of proteins, including p50, p52, RelA (p65), RelB, and c-Rel, which combine to form different dimers to regulate changes in gene expression.^207^ In their inactive form, NF*κ*B dimers are sequestered in the cytoplasm through binding to the inhibitor of NF*κ*B (I*κ*B).^208^ Upon activation by cytokines or cellular stress, the upstream kinases, I*κ*B kinase *α* and *β* (IKK*α*/*β*), together with NF*κ*B essential modulator (NEMO/I*κ*B kinase *γ*) phosphorylate I*κ*B.^209^ Phosphorylation of I*κ*B leads to its degradation, and nuclear translocation of NF*κ*B, where they bind to regulatory elements in the promoter region of their target genes. Direct interactions between AHR and RelA were reported to reciprocally repress both pathways.^210^^,^^211^ Other studies, however, provide evidence that activated RelA/p50 increases *AHR* expression levels and that AHR ligation enhances NFκB-dependent gene regulation.^212^^,^^213^ AHR has been reported to interact with RelB to regulate *interleukin 8* (*IL-8*) gene and chemokine levels,^214^ contributing to AHR’s role in inflammation.

The Janus kinase/signal transducer and activator of transcription (JAK/STAT) pathway is an intracellular signaling cascade relaying extracellular signals from cytokines and growth factors to cellular proteins that regulate cell division, differentiation, immunity, and survival. JAK/STAT signaling is important for proper immune cell signaling and its dysregulation is associated with chronic inflammatory diseases, autoimmunity, and cancer. AHR mRNA levels are increased by activated STATs, including STAT1, STAT3, and STAT5. Type I interferon signaling and subsequent increases in interferon beta levels activate STAT1, resulting in the recruitment of STAT1, STAT2, and interferon regulatory factor 9 that are collectively referred to as interferon stimulated gene factor 3, which binds to and positively increases AHR levels in astrocytes (Fig. 7E).^215^ In addition to regulating AHR expression, STAT1 induction of interferon gamma causes elevated IDO1 levels, thereby stimulating AHR signaling through increased AHR ligand concentrations.^216^^,^^217^ AHR’s effects on STAT signaling vary depending on the cellular conditions and cytokine. AHR can either stimulate or inhibit JAK/STAT signaling through a variety of mechanisms, including altering their phosphorylation status.^215^^,^^218^^,^^219^ AHR stimulates the expression of suppressor of cytokine signaling 2 and 3.^220^^,^^221^ AHR also negatively regulates type I interferon (IFN) signaling by increasing PARP7 levels.^222^ PARP7 represses type I IFN signaling at multiple levels by affecting TANK binding kinase 1 phosphorylation, interferon regulatory factor 3 activity, and STAT1 levels.^222^, ^223^, ^224^ In addition, AHR interacts with other transcription factors, retinoic acid receptors, estrogen receptors, and cell cycle regulators E2F and retinoblastoma protein to modulate their activity.^17^

#### 4 Nongenomic aryl hydrocarbon receptor signaling

**Fig. 8:**
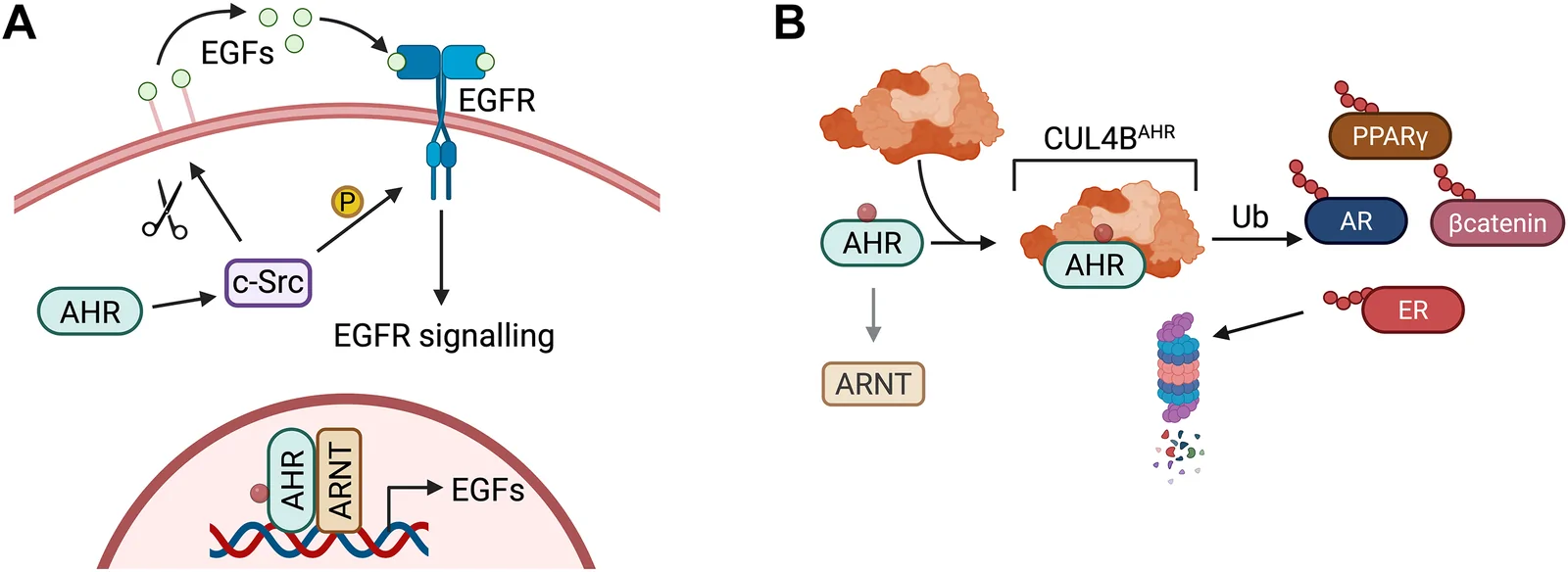
Noncanonical nongenomic AHR signaling. (A) AHR upregulates transcription of EGFs and cytosolic AHR interacts with soluble tyrosine kinase (c-Src) that facilitate phosphorylation of EGFR and shedding of membrane-bound EGFs, activating EGFR signaling. (B) Generation of the E3 ubiquitin ligase complex, CUL4B^AHR^, upon inhibition of ARNT dimerization. The CUL4B^AHR^ complex ligate ubiquitin tails to its protein targets facilitating proteasomal degradation. AR, androgen receptor; PPAR*γ*, peroxisome proliferator activated receptor gamma. Created in BioRender. Matthews, J. (2026) https://BioRender.com/oyiwhoy.

AHR can regulate cell signaling through several nongenomic pathways (Fig. 8). The ubiquitin proteasome system is crucial for maintaining cellular homeostasis through targeted protein degradation.^225^ In this highly regulated process, an E1 activating enzyme transfers a ubiquitin molecule to an E2 ubiquitin-conjugating enzyme. E3 ubiquitin protein ligases then help the E2 enzymes recognize and conjugate one or more ubiquitin molecules to their target proteins, initiating their degradation by the proteasome.^226^ AHR was reported to function as an E3-ligase as part of a CUL4B-based ubiquitin ligase complex, resulting in the target degradation of estrogen receptor *α* (ER*α*), androgen receptor, peroxisome proliferator-activated receptor *γ,* and *β*-catenin.^160^^,^^227^^,^^228^ The E3 ubiquitin ligase function of AHR is independent of ARNT.^229^ More recently, AHR was found to restrain type I IFN signaling by facilitating the ubiquitin-mediated proteasome degradation of stimulator of interferon gene in bladder cancer.^230^

Furthermore, cytosolic AHR interacts with and activates soluble tyrosine kinase, subsequently promoting phosphorylation of epidermal growth factor receptor (EGFR) and shedding of membrane-bound EGFs, thus activating downstream EGFR signaling.^231^^,^^232^ Nuclear AHR induces the expression of EGFR ligands to stimulate EGFR signaling.^233^ In breast cancer cells, reactive oxygen species trigger AHR activation, promoting the increase of the EGFR ligand, amphiregulin, regulating protumorigenic tumor microenvironment (TME).^234^ FICZ and B[*a*]P induced amphiregulin and epiregulin associated with metastatic phenotype of head and neck squamous cell carcinoma cells.^235^ Cytosolic AHR influences calcium-dependent signaling transduction pathways and protein kinase C activity by regulating intracellular calcium levels in response to the PAH, pyrene.^236^ Collectively, these diverse noncanonical and nongenomic actions reinforce the key role of AHR in cellular responses and likely contribute to cell-, tissue-, and context-specific AHR regulation.

### D Aryl hydrocarbon receptor-ligand structure

**Fig. 9:**
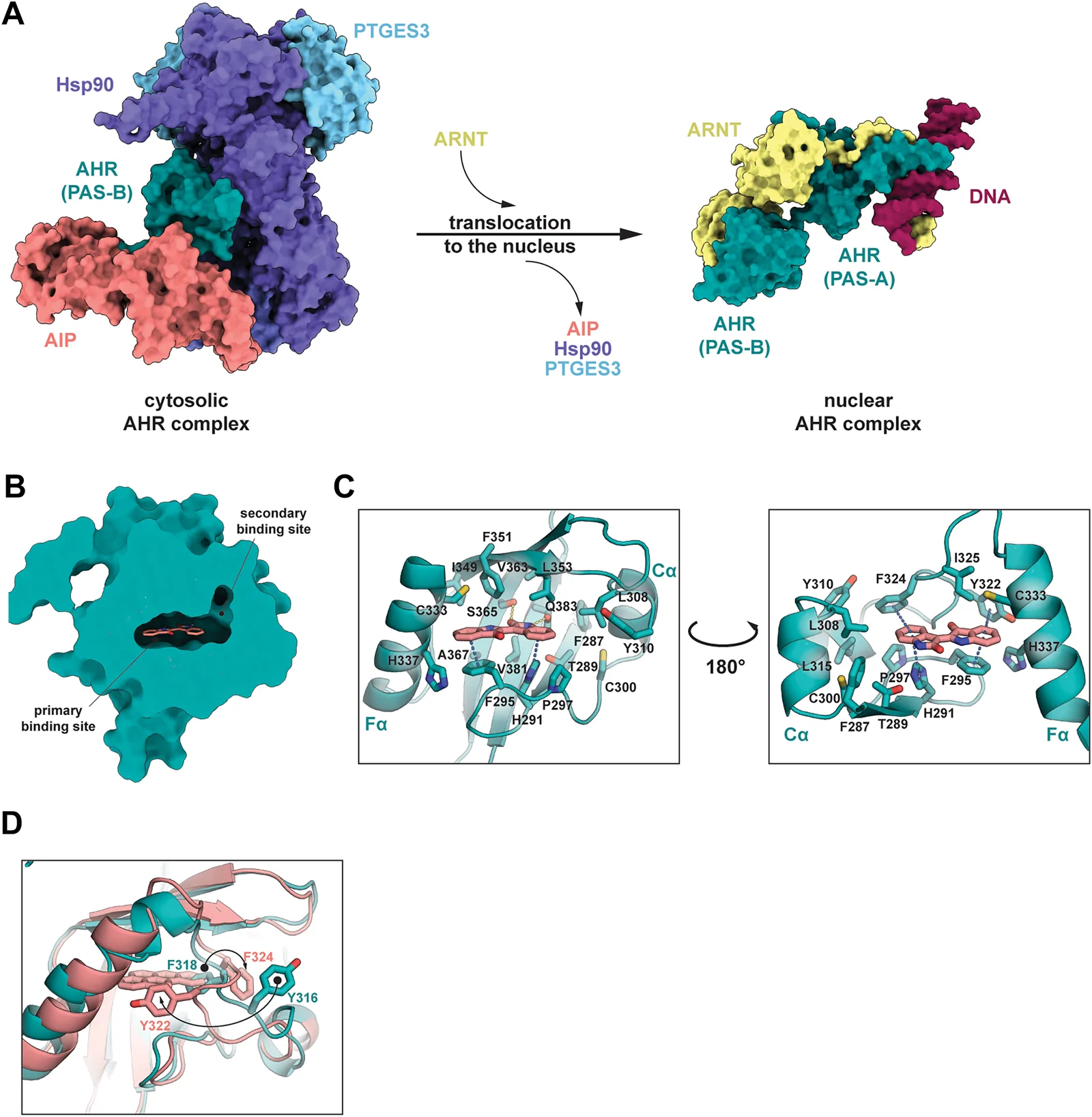
(A) The structures of cytosolic and nuclear AHR complexes. The cryo-electron microscopy (cryo-EM) structure of the cytosolic complex (on the left) consists of Hsp90 (violet), PTGES3 (light blue), AIP (salmon) and AHR (teal) (PDB: 7Y04); whereas the X-ray structure of the nuclear AHR complex (on the right) consists of AHR (teal), ARNT (yellow), and DNA (red) (PDB: 8XSB). (B) The shape and location of the AHR binding site in the PAS-B domain. The AHR PAS-B domain is shown in a teal surface representation, ligand (indirubin)—as salmon sticks. (C) The key binding site residues and ligand-AHR interactions observed. Indirubin and AHR residues within 3 Å from the ligand are shown as sticks. Hydrogen and main chain atoms are omitted for clarity. Yellow and blue dashed lines indicate hydrogen and *π*–*π* stacking interactions, respectively. (D) The D*α*–E*α* loop residue rearrangement upon ligand binding. The ligand (benzo[*a*]pyrene) atoms and sidechains of the residues changing the orientation are shown as sticks. Arrows indicate the rearrangement direction upon ligand binding. The apo *Mm*PAS-B (PDB: 8H77) is shown in teal, the ligand-bound *Hs*PAS-B (PDB: 8QMO) in salmon.

Recently, several crystal and cryo-electron microscopy structures of AHR have been solved. These structures will undoubtedly advance AHR drug identification programs. Because of its multidomain architecture, the full-length crystal structure of AHR is not yet available, although individual domains have been resolved. The first solved structure was the PAS-A domain homodimer (Protein Data Bank [PDB]: 4M4X),^237^ followed by PAS-A–ARNT heterodimers bound to DNA (PDB: 5NJ8, 5V0L).^238^^,^^239^ These structures correspond to the partial nuclear AHR–ARNT–DNA complex formed after nuclear translocation. The first structures of the AHR PAS-B domain were reported by 2 separate groups in 2022.^240^^,^^241^ The AHR PAS-B domain obtained for the *Drosophila* AHR was solved in multiple forms: the AHR PAS-B domain in both apo and ligand-bound states (PDB: 7VNA, 7VNH), and the AHR-ARNT PAS-B heterodimer (PDB: 7VNI).^241^ More recently, structural information has emerged for cytosolic AHR complexes. The AHR–Hsp90–PTGES3–XAP2 complex (PDB: 7Y04)^242^ provided structural information on the bridge motif (P269–K277) connecting PAS-A and PAS-B. The structures of the AHR–Hsp90–AIP (PDB: 7ZUB)^240^ and AHR–Hsp90–PTGES3 (PDB: 8H77)^242^ complexes provided the first experimental data on the AHR ligand-binding domain in a cytosolic complex (Fig. 9A). In these, the mouse AHR PAS-B domain was observed in its *apo* form, whereas the human AHR–Hsp90–AIP complex was crystallized with indirubin bound to the ligand binding domain.

#### 1 Aryl hydrocarbon receptor ligand-binding domain

The AHR ligand-binding pocket is buried and located between helices C*α* and F*α* (Fig. 9, B and C); nonetheless, all elements of the PAS-B secondary structure contribute to its architecture.^240^ The cavity is predominantly hydrophobic (71% of residues), comprising 8 aromatic (26%) and 14 aliphatic (45%) residues. All the ligand interactions for which the binding mode has been resolved (see Table 2), occur mainly near the F*α* helix—the primary binding site, whereas the region adjacent to the C*α* helix (secondary binding site) remains unoccupied (Fig. 9B). Such a bipartite ligand binding site arrangement explains the high binding promiscuity of AHR, which can be bound and activated by a vast array of compounds, ranging from small indole derivatives with a molecular weight below 200 Da to large molecules of around 600 Da, such as bilirubin.^243^ The unique spatial arrangement of 6, mostly hydrophobic, residues on both faces of the planar primary ligand binding site (Fig. 9, C and D) provides a clear explanation for the distinct preference of AHR for compounds displaying a planar structure.

**Table 2:** AHR ligand-binding domain-containing structures

Ligand	Cytosolic Complex (AHR-Hsp90)	Nuclear Complex (AHR-ARNT-DNA)	AHR PAS-B
apo	8H77∗ (+PTGES3; +XAP2) 7Y04 (+ PTGES3)	5V0L 5NJ8 7VNI (-DNA)	7VNA
Benzo[*a*]pyrene	8QMO (+XAP2)	8XS8	
Indirubin	7ZUB (+XAP2)	8XSB	
Tapinarof		8XS6	
FICZ		8XS7	
*β*-Naphthoflavone		8XS9	
Indigo		8XSA	
*α*-Naphthoflavone			7VNH

∗ Four-character alphanumeric code refers to unique Protein Data Bank Identifier (PDB ID).

Thus far, the binding modes of several AHR agonists have been determined. Indirubin and B[*a*]P^68^^,^^243^ are the ligands for which their binding modes in both the cytosolic and nuclear AHR complexes have been solved, whereas the binding modes of tapinarof, FICZ, *β*-naphthoflavone, and indigo have been determined in the nuclear AHR complex (see Table 2). There are no pronounced differences in ligand and PAS-B domain conformation between the cytosolic and nuclear complexes of the indirubin and B[*a*]P. The ligand root mean square deviation between both complexes are 0.51 and 0.60 Å for indirubin and B[*a*]P, respectively, and the AHR binding domain root mean square deviation are 0.64 and 0.72 Å, respectively. Moreover, the type of agonist bound does seem to affect the geometry of the PAS-B domain only slightly, as the root mean square deviation of the PAS-B domain backbone atoms is in the range between 0.30 and 0.88 Å for the agonist-bound complexes.

All the agonists with a verified binding mode are binding in the primary binding site and are engaged in hydrophobic and aromatic contacts with H291, F295, P297, Y322, F324, I325, C333, H337, I349, F351, L353, and/or V381 (human AHR numbering). In addition to these nonpolar interactions, several agonists are involved in hydrogen bonding interactions with T289, S365, and Q383 side chains and/or G321 main chain, which greatly contribute to the binding affinity.^243^ The key interactions that are formed upon ligand binding are depicted in Fig. 9D (indirubin as an example).

#### 2 Ligand binding induced structural changes

Although the type of agonist bound does not seem to play a crucial role in PAS-B geometry, the ligand binding event itself does change the conformation of the AHR PAS-B domain. It is believed that these ligand-induced conformation changes mediate AHR regulation.^243^ The conformational changes in the AHR PAS-B domain are transmitted through HSP90 to the N-terminal region of AHR, exposing the nuclear localization signal to the importin complex and thereby triggering nuclear translocation. The structure alignment indicates that no substantial changes in the secondary structure of the PAS-B domain are introduced upon ligand binding. However, the loop connecting helices D*α* and E*α* (the D*α*–E*α* loop) does rearrange and is thought to play a central role in receptor activation. In apo *Mm*PAS-B (PDB: 8H77), which likely represents the inactive state, the loop adopts a different conformation compared with ligand-bound PAS-B structures (eg, PDB: 8QMO), considered to represent the active form. Upon ligand binding, residues of the D*α*–E*α* loop that contact the ligand are displaced by several Å (see Fig. 9D). This displacement appears to stabilize a specific conformation of the loop. The same loop also serves as a docking surface for the C-terminal extension that links PAS-B to the transactivation domain, as well as interacts with the cochaperone AIP. Therefore, it is thought that ligand-dependent rearrangement of the D*α*–E*α* loop might play a role in numerous processes, including ligand recognition and binding, protein–protein interaction, and possibly receptor activation.^243^ Further high-resolution structural and functional studies are required to describe the involvement of the D*α*–E*α* loop in the regulation of AHR function in more detail.

## III Aryl hydrocarbon receptor in inflammation and innate and adaptive immunity

AHR is important for the differentiation and function in many innate and adaptive immune cells. There are several excellent reviews that describe the function of AHR in immune cells,^244^, ^245^, ^246^ so we only provide a brief overview here.

### A Role of aryl hydrocarbon receptor in innate immune cells

Macrophages represent a heterogeneous population of antigen presenting cells that are involved in all phases of the immune responses. Macrophages exhibiting M1 phenotype are characterized by high cytotoxic potential and are considered antitumorigenic, whereas M2-polarized macrophages are associated with immune suppression and tumor progression in part through suppression of cytotoxic T cell function.^247^ *Ahr* loss enhanced inflammatory cytokine levels from M1 polarization of macrophages and from macrophages isolated from tumor-bearing or septic mice.^248^, ^249^, ^250^ In M2-polarized macrophages, *Ahr* deficiency reduced IL10 and arginase levels, suggesting reduced M2 polarization.^248^ Tumor-associate macrophages (TAMs) present in the TME are implicated in cancer initiation, progression, metastasis, and responses to therapy. As with in vitro characterized macrophages, the functions of TAMs are also impacted by AHR signaling. Tryptophan metabolites generated from IDO1 and TDO activated AHR to promote an M2-like immunosuppressive phenotype in a melanoma model.^251^ Loss of AHR in TAMs improved their inflammatory phenotype, resulting in reduced tumor growth in pancreatic cancer.^252^ Overall, AHR favors an immunosuppressive macrophage phenotype, reducing inflammation and antitumor immunity.

Dendritic cells (DCs) act at the interface between the innate and adaptive immune systems and are crucial for balancing immunity and tolerance, and for controlling T cell responses.^253^ AHR activation by FICZ in the presence of IL4 and tumor necrosis factor *α* promoted the differentiation of monocytes to DCs in favor of macrophages.^254^ Treatment with the AHR agonist, VAF347, prevented the differentiation of CD34^+^ cells to Langerhans DCs and inhibited the function of human monocyte-derived DCs to induce T cell proliferation and cytokine production.^255^^,^^256^ The tryptophan metabolite, 3-hydroxyanthranilic acid, was shown to stimulate tolerogenic DCs via AHR activation.^257^ Thus, AHR influences DC antigen presentation and T cell priming. AHR activation favors a tolerogenic DC phenotype, but this is ligand- and context-dependent.^258^

### B Innate lymphoid cells

Innate lymphoid cells (ILCs) are derived from common lymphoid progenitors and can be divided into 3 groups (ILC1–3) with a total of 5 subsets: natural killer (NK) cells, ILC1s, ILC2s, ILC3s, and lymphoid tissue inducer cells. ILC1s comprise 2 subsets of T-bet expressing NK cells and type 1 ILCs. ILC1s exert rapid and first-line responses to viral and bacterial infections.^259^ ILC2s are characterized by GATA-binding protein 3 expression and are found in the lungs, gastrointestinal tract, and skin.^260^ ILC2s have a role in tissue homeostasis, including allergic diseases, and parasite elimination. Group 3 ILCs (ILC3s) are defined by their retinoic acid related orphan receptor gamma t (ROR*γ*t) expression and divided into NKp46+ ILC3 and NKp46− ILC3. ILC3s are abundant at intestinal mucosa, skin, and lungs. They produce IL22 and IL17, which promote barrier repair, antimicrobial production, and recruitment of other immune cells.^261^ Lymphoid tissue inducer subset is also dependent on ROR*γτ* and crucial for secondary lymphoid organogenesis during embryogenesis.^262^ AHR was reported to be required for the maintenance of liver resident NK cells,^263^ and *Ahr* loss in NK cells reduced their migratory, cytolytic and antitumor activity, supporting a role of AHR in NK cell function.^264^^,^^265^ Even though AHR is expressed in ILC2s,^266^ its function in ILC1s and ILC2s is not known. In contrast, AHR regulates ILC3s differentiation and proliferation.^267^^,^^268^ AHR also promotes IL22 production through its regulation of ROR*γτ* in ILC3s, which is essential for clearance of intestinal bacterial infections.^267^^,^^269^

### C Role of aryl hydrocarbon receptor in adaptive immune cells

#### 1 CD4^+^ T helper cells

CD4^+^ T cells and CD8^+^ T cells constitute the majority of T-lymphocytes. CD4^+^ T cells have several functions, such as activation of innate immune cells, B cells, and cytotoxic T cells, but also suppression of immune responses.^270^ There are several different CD4^+^ T helper cells, including T-helper 1 (Th1) cells, T-helper 2 (Th2) cells, T-helper 17 (Th17) cells, T-helper 22 (Th22) cells, follicular helper T cells, T-regulatory cells (Tregs), and the regulatory type 1 (Tr1) cells. AHR promotes the differentiation and affects the function of Th17, Th22, Tregs, and Tr1 cells.^191^^,^^271^, ^272^, ^273^ AHR supports Th17 cell differentiation by activating STAT3-dependent regulation of Aiolos, which inhibits early Th17 differentiation, and by repressing STAT1 and STAT5 activation. However, the role of AHR in Th17 cell differentiation is ligand selective, with FICZ stimulating and TCDD inhibiting differentiation.^272^ AHR activation with TCDD in the presence of transforming growth factor *β* stimulates Treg differentiation through small mothers against decapentaplegic homolog-dependent regulation of transcription factor forkhead box P3.^274^ Tumor-generated kynurenine activates AHR to drive Treg cell generation and tolerogenic TAMs in B16 mouse melanoma tumors.^251^ Tr1 cells are immunosuppressive cells that lack forkhead box P3 and produce high levels of IL10. These cells are crucial immune regulators that maintain tolerance, prevent autoimmunity, and suppress inflammation. AHR is required for the maintenance of differentiation of Tr1 cells.^191^^,^^274^^,^^275^

#### 2 CD8^+^ T cells

AHR differentially regulates the fate of CD8^+^ T cells. *Ahr* loss in mouse CD8^+^ T cells reduces CD8^+^ granzyme-B-producing CD69^+^CD103^+^ T resident memory (T_RM_) cells, whereas AHR activation promotes the differentiation of human intestinal intraepithelial CD8^+^ T cells to T_RM_ cells as well as granzyme B production.^276^ However, AHR reduces CD8^+^ T effector cells during influenza A infection.^277^ Kynurenine activated AHR induces the expression of the immune checkpoint protein, programmed cell death protein 1 (PD-1) on CD8^+^ T cells, promoting T cell exhaustion, which was prevented with exogenous AHR agonist treatment in a mouse melanoma cancer model.^278^ Consistent with these data, AHR regulates the expression of other immune checkpoint proteins, including programmed cell death protein ligand 1 (PDL1), PDL2, cytotoxic T-lymphocyte associated protein 4, lymphocyte-activation gene 3, and V-set domain-containing T cell activation inhibitor 1 (B7-H4).^217^^,^^279^, ^280^, ^281^ More studies are needed to better understand the opposing outcomes that AHR has on CD8^+^ T cell responses.

## IV Aryl hydrocarbon receptor as a therapeutic target

AHR has emerged as a critical physiological regulator of metabolic homeostasis, lipid metabolism, inflammation and immune responses. This has led to significant interest in pursuing AHR activators as therapeutic options for inflammatory diseases and autoimmune disorders.^282^ The contrasting effects of AHR in cancer cell intrinsic malignant properties and antitumor immunity, and how AHR signaling should be targeted (ie, agonist vs antagonist) is tumor type dependent.^3^

### A. aryl hydrocarbon receptor as a target for metabolic disorders

One of the striking and mechanistically unresolved outcomes of TCDD toxicity in rodents is the lethal wasting syndrome.^283^^,^^284^ A single dose of TCDD induces a lethal starvation-like syndrome, characterized by reduced gluconeogenesis, liver damage, steatohepatitis, dyslipidemia, and lethality.^284^^,^^285^ These findings implicate AHR in energy metabolism, fatty liver disease and metabolic syndrome. Indeed, transgenic AHR mice develop steatosis, whereas *Ahr*^*−/−*^ mice are resistant to the effects of TCDD.^286^^,^^287^ At nonlethal doses, TCDD induces insulin resistance and the development of metabolic dysfunction, fatty liver disease (previously referred to as nonalcoholic fatty liver disease), which could develop into metabolic dysfunction-associated steatohepatitis, liver fibrosis, and cancer.^288^^,^^289^ Epidemiological studies have linked TCDD and AHR activating environmental pollutants to diabetes, insulin resistance, and metabolic syndrome.^290^, ^291^, ^292^, ^293^, ^294^ Low dose TCDD promotes high fat diet (HFD)-induced obesity in mice, whereas *Ahr*^*−/−*^ mice are resistant to the negative impact of HFD-induced weight gain. Moreover, congenic C57BL/6 expressing *Ahr*^*d*^ allele are protected from HFD-induced obesity and metabolic dysfunction compared with wild-type mice expressing *Ahr*^*b1*^ allele.^295^ Adipose-specific depletion of AHR is sufficient to protect from HFD-induced obesity and metabolic dysfunction, with the protective effects more profound in female compared with male mice.^296^ Mice deficient in IDO1 are resistant to diet-induced obesity, suggesting that kynurenine stimulates AHR and promotes obesity. In line with this, the AHR antagonists, *α*-naphthoflavone, resveratrol and CH223191 reduce obesity, and ameliorate metabolic dysfunction, fatty liver disease.^297^^,^^298^ However, dietary exposure to AHR proligand I3C or AHR agonist, indigo, protects against diet-induced obesity, liver toxicity, liver steatosis, and inflammation.^299^, ^300^, ^301^ Moreover, emerging evidence supports a role of the gut microbiota in liver pathogenesis.^302^ HFD diet reduces the levels of AHR ligands, tryptamine and indole-3-acetate. Indole-3-acetate was further shown to attenuate hepatocyte inflammatory responses and reduce the expression of genes regulating lipogenesis.^303^ Supplementation with FICZ or AHR ligand producing *Lactobacillus* results in altered glucose metabolism and liver steatosis.^304^ Because both AHR activation and inhibition protect against obesity and its associated metabolic dysfunction in rodents, more studies are needed to determine if targeting AHR is a feasible approach to treat obesity, liver steatosis, and metabolic disorders.

### B Aryl hydrocarbon receptor as a therapeutic target for intestinal inflammatory diseases

#### 1 Ulcerative colitis and Crohn’s disease

Inflammatory bowel diseases (IBDs), such as ulcerative colitis and Crohn’s disease, are frequently associated with dysregulated innate and T cell immune responses, arising from a disruption in host-microbiota homeostasis driven by genetic susceptibility and environmental factors. Current treatment strategies include corticosteroids, aminosalicylates, and immunosuppressants as well as biologics targeting tumor necrosis factor *α*, and JAK integrins.^305^ However, variable efficacy, side effects, and high costs constrain their clinical use,^306^ supporting the need for new therapies. AHR has emerged as a potential therapeutic target for IBD based on promising findings in several preclinical models.^307^^,^^308^

In the gastrointestinal tract, many of the anti-inflammatory effects of AHR activation are attributed to the induction of IL22, maintenance of intraepithelial lymphocytes and ILC3s, as well as the modulation of effector cells.^268^^,^^309^ In a dextran sodium sulfate-induced colitis mouse model, AHR deficiency or loss of the ability of AHR to bind AHREs exacerbates colonic inflammation.^310^^,^^311^ *Ahr*-deficient mice and selective loss of *Ahr* in intestinal epithelial cells, as well as CYP1A1-overexpressing mice with reduced endogenous AHR ligand levels, are sensitive to *Citrobacter rodentium* intestinal inflammation.^169^^,^^312^ Loss of *Ahr* increases the risk of colorectal cancer because of chronic intestinal inflammation in mice.^313^ Consistent with these findings, AHR agonists, increases in levels of AHR ligand-producing bacteria, and diets rich in tryptophan alleviate the severity of colitis in both dextran sodium sulfate- and trinitrobenzene sulfonic acid-induced wild-type and human mouse models.^314^, ^315^, ^316^ Caspase recruitment domain family member 9, a susceptibility gene for IBD, promotes recovery from colitis by stimulating IL22 production. *Card9*^*−/−*^ mice are more susceptible to intestinal inflammation because of reduced AHR ligand-producing microbiota, which is reduced by AHR ligand producing *Lactobacillus* bacterial strains or by treatment with AHR agonists.^317^

The strongest clinical evidence for targeting AHR to treat intestinal inflammation arises from studies using indigo naturalis. Indigo naturalis (Qing-Dai) is an indigo-blue powder derived from the stems and leaves of Acanthaceae plants that has been used for years as a natural remedy to treat inflammatory diseases, including IBD. Indigo naturalis composition is regulated in China to contain more than 2.0% indigo and more than 0.13% indirubin, both of which are AHR agonists.^318^ Recent clinical trials support the effectiveness of indigo naturalis for the treatment of ulcerative colitis, especially for refractory cases.^319^, ^320^, ^321^, ^322^ The combination of indigo naturalis and curcumin showed clinical promise for patients with moderate to severe ulcerative colitis, even for those resistant to biologics and/or corticosteroids.^323^ Two recent reviews concluded that the therapeutic effects of indigo naturalis in ulcerative colitis are closely linked to its ability to regulate the AHR receptor, exert anti-inflammatory effects, modulate intestinal flora, and restore the intestinal barrier integrity.^324^^,^^325^ However, there are some concerns regarding adverse events, such as pulmonary hypertension, as well as quality control issues, and insufficient pharmacokinetic data. Regardless, these studies support pursuing AHR signaling as a therapeutic strategy for ulcerative colitis. In line with this, AT177 is an orally active AHR agonist and investigational drug developed by Azora Therapeutics that is being considered for the treatment of ulcerative colitis.^326^ DMVT-506 and AQ312 are AHR agonists developed by Dermavant and Aquilon, respectively, that are currently under preclinical investigation for the treatment of inflammatory bowel disease.^327^

#### 2 Coeliac disease

Coeliac disease is a chronic inflammatory disease triggered by dietary gluten, which is present in grains including wheat, rye, and barley. The activated immune response damages the intestinal lining, preventing nutrient absorption and causing symptoms such as diarrhea, fatigue, bloating, and weight loss.^328^ AHR expression levels were reduced in the intestinal mucosa of patients with active coeliac disease.^329^ FICZ protects against poly I:C-induced intestinal enteropathy by reducing inflammatory cytokine levels and cytotoxic factors.^329^ Nonobese diabetic mice expressing DQ8, a coeliac disease susceptibility gene, placed on a high-tryptophan diet, and given AHR ligand producing *Lactobacillus reuteri* or treated with FICZ, showed decreased immunopathology exposed to gluten.^330^ These findings suggest that AHR could represent a new therapeutic strategy for this disease.

### C Lung inflammation

Because of ongoing inflammatory processes, lung diseases such as asthma and chronic obstructive pulmonary disease (COPD) are among the most common medical conditions in the world. Asthma affects the bronchial tubes, where the airways overreact to external factors such as smoke, air pollution, and allergens. COPD is another inflammatory disease that affects both the airways and lung tissue. Cigarette smoke is the leading cause of COPD.

#### 1 Asthma

Preclinical studies in animal models provide support for a role for AHR in asthma.^331^ *Ahr* deficiency causes increased airway infiltration of eosinophils and lymphocytes with elevated production of IL4 and IL5 in an ovalbumin-induced allergic asthma model. Consistent with these findings, *Ahr* loss enhances, whereas AHR ligand activation suppresses lung inflammation in a cockroach allergen asthma model.^332^ However, *Ahr* loss does not affect the inflammatory response in an irritant-induced asthma model,^333^ suggesting that the role of AHR in regulating asthma outcomes varies with different etiological agents. TCDD alleviates noneosinophilic airway inflammation by decreasing neutrophil recruitment and Th17 cytokine expression.^334^ *Ahr*-deficient mice show increased immune cell recruitment, airway hyperreactivity, epithelial injury, inflammation, and fibrosis after ozone exposure.^335^ However, epithelium-derived thymic stromal lymphopoietin, IL25, and IL33, which are associated with Th2 immune responses in severe allergic asthma, are upregulated by diesel exhaust in an AHR dependent manner.^336^ In ovalbumin-induced asthma model, indeno[1,2,3-cd]pyrene increases lung Th2 immune responses through AHR activation.^337^ Overall, these data suggest that AHR has opposite responses in different asthma models, leading to both beneficial and deleterious outcomes in asthma.^338^

#### 2 Chronic obstructive pulmonary disease

Emerging experimental evidence supports that AHR mitigates the development of COPD by reducing pulmonary inflammation. Human cigarette smokers and mice exposed to cigarette smoke have high pulmonary levels of macrophages, CD8^+^ T-lymphocytes and neutrophils.^339^^,^^340^ Diesel exhaust particulate and PAHs, such as B[*a*]P, trigger lung inflammation by activating AHR, which increases Cxcl1, Il8, and Il6 cytokine levels, attracting neutrophils to the airways.^341^, ^342^, ^343^ The AHR activating ligands in cigarette smoke suggest that AHR may promote COPD; however, *Ahr*-deficient mice showed increased pulmonary neutrophil infiltration after acute smoke exposure and heightened pulmonary inflammation after chronic exposure.^331^^,^^344^ In agreement with these findings, treatment with the AHR antagonist, CH223191, significantly enhanced airway neutrophilia.^345^ FICZ activated AHR promoted macrophage uptake of neutrophils through a nongenomic AHR signaling mechanism involving IL10 and JAK/STAT signaling.^346^ Taken together, these results support a role for AHR modulators as therapeutic agents to treat chronic inflammatory lung diseases such as COPD.

### D Inflammatory skin diseases

Emerging evidence supports that activation of AHR is beneficial for the maintenance of healthy skin and for dampening skin inflammation.^347^^,^^348^ AHR is highly expressed in keratinocytes but also in melanocytes, fibroblasts, Langerhans cells and *γδ* T cells.^349^ UV generation of FICZ plays an important role in maintaining healthy skin.^350^ The skin is also exposed to many exogenous AHR ligands, such as PAHs from air pollution, and indole-derived ligands generated from commensal skin microbiota.^351^^,^^352^ AHR activation with coal tar, which contains high levels of PAH, reduces inflammation and restores barrier function in atopic dermatitis and psoriasis. The actions of coal tar were proposed to be due to AHR-regulated activation of the NFE2L2-mediated antioxidative stress pathway.^353^ In an imiquimod-induced mouse model of skin inflammation, FICZ reduced inflammation, whereas *Ahr* loss exacerbated disease outcomes.^347^ Difamilast, a phosphodiesterase 4 inhibitor, alleviates atopic dermatitis symptoms and inflammation via the AHR-NFE2L2 signaling axis.^354^

These studies led to the discovery of tapinarof (DMVT-505, GSK2894512, WBT-1001, Benvitimod), the first-in-class AHR agonist that was approved by the FDA as 1% topical cream (VTAMA) treatment for plaque psoriasis in 2022 and atopic dermatitis in 2024.^76^^,^^77^ Tapinarof was developed by Dermavant Sciences, which was recently acquired by Organon.^355^ Tapinarof is a natural stilbene first isolated from gram-negative *Photorhabdus luminescens* bacteria, which infect the gut of nematodes of the genus *Heterorhabditis*.^76^ Upon infection of an insect, *Heterorhabditis* nematodes release *P luminescens* bacteria that kills the insect. It was noted that insects infected with high levels of *P luminescens* did not putrefy (decompose), suggesting that they release chemicals with antimicrobial properties.^356^^,^^357^ Tapinarof (3,5-dihydroxy-4-isopropylstilbene) was isolated and found to activate AHR and NFE2L2 pathways, reducing inflammation in an imiquimod-induced psoriasiform skin lesion model.^78^ Tapinarof-activated AHR downregulates Th2-associated cytokines (IL4, IL5, IL13, and IL31) and Th17-associated cytokines (IL17A and IL17F), which drive the pathogenesis of atopic dermatitis and psoriasis, respectively. Tapinarof increases the levels of epidermal barrier proteins, including filaggrin, loricrin, and involucrin, as well as ceramide-based barrier lipids.^355^ Tapinarof also enhances antioxidant defenses in the skin through activation of the NFE2L2 pathway even in the presence of CH223191, suggesting that it acts as a dual AHR/NFE2L2 agonist.^358^

Tapinarof 1% cream formulations have been studied in several phase 3 trials in patients with plaque psoriasis (PSOARING 1, 2, and 3) or atopic dermatitis (ADORING 1, 2, and 3). In the 12-week controlled PSOARING 1 (NCT03956355) and PSOARING 2 (NCT03983980) trials of adults with mild to severe psoriasis (Physician’s Global Assessment score 2 to 4), significantly more patients achieved clear/almost clear skin (Physician’s Global Assessment score 0 or 1) compared with vehicle.^76^ PSOARING 3 was a long term 40-week intermittent use trial with a high complete disease clearance rate (40.8%), evidence for durable remittive effect and response to intermittent treatment.^359^ Stable disease control was also reported for benvitimod 1% formulation available in China^360^ and for tapinarof 1% cream in 2 clinical trials in Japan.^361^ Tapinarof was well tolerated, with minimal systemic exposure after topical use.^362^ Some of the most common adverse events reported were folliculitis (∼20% of patients), nasopharyngitis and contact dermatitis (5%).^363^ Tapinarof was found to have minimal inhibitory effects on cytochrome P450 enzymes and drug transport proteins.^362^

Tapinarof 1% cream is also effective against atopic dermatitis as evidenced by results from the 8-week ADORING 1 (NCT05014568) and ADORING 2 (NCT05032859) phase 3 trials.^355^ These results prompted further assessment of skin clearance rates, duration of treatment-free intervals, and safety of tapinarof in ADORING 3 (NTC05142774), a phase 3 open-label extension trial with a duration of up to 48 weeks.^364^ Daily application of tapinarof cream 1% resulted in high rates of disease clearance in patients with atopic dermatitis, and clear/almost clear skin was upheld for ∼80 days after discontinuation of treatment.^364^ In December 2024, tapinarof was approved by the FDA for the topical treatment of atopic dermatitis in adults and pediatric patients 2 years of age.^77^ Tapinarof is also being investigated for atopic dermatitis treatment in ongoing clinical trials in China (CTR20231413) and Japan.^365^, ^366^, ^367^

Another emerging AHR agonist in preclinical and clinical trials for the treatment of skin disorders includes AT193, developed by Azora Therapeutics for the treatment of hidradenitis suppurativa, a chronic inflammatory skin disorder, characterized by deep, boil-like lumps, abscesses, and sinus tracts (NCT04989517), and for patients with moderate-to-severe nail psoriasis (NCT05072886).

### E Autoimmune disorders

Seminal studies identifying a role for AHR in modulating inflammation and T cell function have fueled interest in pursuing AHR as a treatment for autoimmune disorders.^271^^,^^272^ These early works led to several studies demonstrating that modulation of AHR signaling alleviates disease progression in experimental autoimmune models and in patients.^368^^,^^369^

#### 1 Multiple sclerosis

Multiple sclerosis is a neuroinflammatory disease of the central nervous system that causes demyelination and neuronal injury.^370^ The prevalence of multiple sclerosis differs by sex (3:1 for females to males) and varies globally, with the highest rates in Europe and the Americas (∼260 cases per 100,000) and lowest rates in African and Western Pacific regions (5 per 100,000).^371^ The adaptive immune system, and specifically CD4^+^ T lymphocytes, plays a central role in multiple sclerosis pathology. Since AHR modulates the differentiation and function of CD4^+^ T cell subsets, it may represent a new therapeutic target for this disease. Administration of ITE or increased tryptophan metabolite production via the IDO1/IDO2 pathway increases DC-expressed transforming growth factor *β* and IL10, leading to the expansion of Treg cells, which reduced neuroinflammation during experimental autoimmune encephalomyelitis (EAE), a mouse model of multiple sclerosis.^372^^,^^373^ Treatment of EAE mice with TCDD, I3C, or DIM prevented disease symptoms by promoting the generation of Tregs and downregulating the levels of Th17 cells.^272^^,^^374^ On the other hand, FICZ treatment favored Th17 cell differentiation, promoting disease progression.^271^ Moreover, resveratrol was reported to trigger apoptosis in activated T cells, reducing the severity of EAE in a mechanism involving AHR and ER activation.^375^ These findings further illustrate the functional differences among AHR ligands and how they modulate effector and regulatory T cells.

Laquinimod (5-chloro-*N*-ethyl-4-hydroxy-1-methyl-2-oxo-*N*-phenyl-1, 2-dihydroquinoline-3-carboxamide), also known as ABR-215062, was identified as an AHR agonist and demonstrated to be effective at reducing EAE disease progression.^376^ Laquinimod was studied in several clinical trials of patients with relapsing-remitting multiple sclerosis and primary progressive multiple sclerosis. In a phase III trial (ALLERGO; NCT00509145), oral administration of 0.6 mg per day laquinimod was well tolerated and significantly reduced disease activity in patients with relapsing-remitting multiple sclerosis.^377^^,^^378^ However, in another study with the same dose (BRAVO; NCT00605215) laquinimod did not differentiate significantly from placebo and was not better than interferon beta 1a therapy.^379^ Because of these inconsistent results, another phase III trial (CONCERTO; NCT01707992) was launched and included doses of 0.6 mg and 1.2 mg per day of laquinimod. Laquinimod failed to slow disease progression in patients with relapsing-remitting multiple sclerosis, and the high dose caused adverse cardiovascular events, resulting in the termination of the trial.^380^ Laquinimod failed to demonstrate a statistically significant effect on brain volume loss in primary progressive multiple sclerosis in the phase II ARPEGGIO trial (NCT02284568).^381^ Recently, PARP7 was reported to suppress type I IFN signaling by reducing STAT1 and STAT2 levels and relieving the severity of EAE symptoms.^224^ These observations warrant further investigation into targeting the AHR-PARP7 axis as a potential treatment strategy for multiple sclerosis.

#### 2 Systemic lupus erythematosus

Systemic lupus erythematosus (SLE) is a chronic autoimmune disease categorized by increased frequencies of autoreactive B cells and T cells, resulting in inflammation and immune-mediated injury to multiple organs.^382^ The impaired clearance of apoptotic cells leads to the accumulation of nuclear material, causing inflammation and autoantibody production. DNA derived from apoptotic cells signals through Toll-like receptor 9 in macrophages to activate AHR and stimulate IL10 production, promoting immune tolerance. Deletion of *Ahr* in the myeloid lineage caused autoimmunity in mice, and treatment with I3C or DIM prevented disease development and reversed established autoimmune pathology.^369^ In line with these findings, studies of monocyte-derived macrophages from patients with SLE revealed that activation of AHR by I3C promotes an anti-inflammatory M2 phenotype and reduces proinflammatory cytokine expression.^383^ Type I IFNs are a primary pathogenic factor in SLE.^384^ Type I IFNs promote the expansion of CD4^+^ T cells into CXCL13^+^ T follicular helper cells and T peripheral helper cells, which help attract B cells to exacerbate disease. CRISPR screen identified AHR as a negative regulator of CXCL13 production by human CD4^+^ T cells. Mechanistically, AHR was found to interact with the AP1 factor JUN to inhibit CXCL13 production to increase disease ameliorating IL22 levels.^385^ Intraperitoneal tapinarof treatment ameliorated lupus phenotypes and reduced inflammation in MRL/lpr mice by inhibiting JAK2-STAT3 signaling and the development of follicular helper T cells.^386^ Collectively, these studies support exploring AHR as a therapeutic target in SLE.

#### 3 Rheumatoid arthritis

Rheumatoid arthritis (RA) is a systemic and chronic inflammatory disease characterized by synovial inflammation and subsequent joint destruction. The rheumatoid synovium consists of CD4^+^ and CD8^+^ T cells, B cells, and macrophages.^387^ Higher AHR mRNA and protein levels are observed in RA synovial tissue. Cigarette smoking and dioxin increase proinflammatory cytokine levels in synoviocytes from patients with RA.^388^^,^^389^ AHR agonists, TCDD, and FICZ, increase arthritis severity by promoting Th17 cell polarization and infiltration into the inflammatory joints and draining lymph nodes of arthritic transgenic mice carrying the human HLA-DRB1 shared epitope coding allele associated with RA compared with control treatment.^390^ The AHR antagonist GNF351 attenuates proinflammatory cytokine signaling in primary human fibroblast-like synoviocytes by inhibiting recruitment of AHR to *IL1B* and *IL6* genes.^391^ AHR loss in T cells inhibited the generation of Th17 cells and inflammation in a collagen induced arthritis mouse model.^392^ The AHR modulator resveratrol, which is reported to both inhibit and activate AHR, attenuated clinical parameters in collagen induced arthritis mice, which were associated with markedly reduced serum levels of proinflammatory cytokines. However, indole-3-pyruvate inhibits the differentiation of Th17 cells and promotes the differentiation of Treg cells in vitro and reduced disease severity of collagen induced arthritis in rats.^393^ AHR modulation may represent a new approach for the prevention and treatment of RA.

### F Viral, bacterial, and fungal infections

AHR’s role in regulating innate and adaptive immune cell function has led to interest in pursuing AHR modulation to treat pathogen infections.^394^^,^^395^

#### 1 Influenza

Several studies have reported a correlation between respiratory infections and AHR activation.^351^ In mouse models, TCDD exacerbated inflammatory lung disease by affecting follicular helper T cells and CD8^+^ T and B cell function during influenza A virus infection.^396^, ^397^, ^398^ TCDD-activated AHR independently enhances neutrophil recruitment and increases inducible nitric oxide synthase expression in the lungs, suggesting that AHR balances protective and excessive inflammation during influenza A infection.^398^, ^399^, ^400^ The metabolic stability of AHR agonists (TCDD vs FICZ) and treatment time produced distinct immunomodulatory effects depending on activation duration and target cells.^401^ AHR activation limited viral infection of influenza A virus, vesicular stomatitis virus, Newcastle disease virus, Sendai virus, encephalomyocarditis virus, and herpes simplex virus-1 in mouse embryonic fibroblasts through increased PARP7 levels. AHR activation by kynurenine reduced interferon beta levels, resulting in increased viral replication, which was dependent on PARP7.^222^ PARP7 is an important regulator of innate immune responses by its direct actions of TANK binding kinase 1, interferon regulatory factor 3, and STAT1.^222^^,^^223^ ARNT directly interacts with the influenza H5N1 polymerase acidic protein, reducing viral polymerase activity and limiting viral replication, suggesting that AHR activation can indirectly affect influenza infection through binding to ARNT.^402^ Overall, AHR activation modulates influenza infection through immune regulation and direct effects on viral replication machinery.

#### 2 Coronavirus

Coronaviruses, such as severe acute respiratory syndrome coronavirus 2, are positive sense single-stranded RNA viruses capable of infecting a wide range of hosts. Several coronaviruses increase AHR levels and exploit AHR signaling to evade the host immune system and facilitate viral replication.^403^, ^404^, ^405^ Pharmacological inhibition of AHR with CH223191 repressed virus yield, reduced mucin production and ameliorated disease phenotypes.^403^^,^^404^^,^^406^, ^407^, ^408^ Coronavirus activation of AHR contributed to the upregulation of PARP7 independently of IDO1 and host tryptophan metabolism.^405^ AHR-dependent increases in PARP7 resulted in reduced type I IFN responses, which promoted maximal viral replication.

#### 3 Other viruses

AHR modulation of adaptive and innate immune cells, kynurenine metabolism, and AHR suppression of type I IFN signaling have been implicated in playing a role in the infection of several other viruses, including Epstein-Barr virus, HIV, Zika virus, and herpes virus.^409^, ^410^, ^411^, ^412^ Collectively, these findings identify AHR as a critical host factor in viral infections and support its further development as a novel antiviral therapy.

#### 4 Bacterial and fungal infections

Emerging studies support a significant role for AHR in the pathogenesis of bacterial, parasitic and fungal infections. AHR is protective against *C rodentium* infection by reducing inflammation and improving epithelial barrier integrity. Similar protective effects of AHR activation have been observed during *Salmonella* and *Escherichia coli* infections.^413^^,^^414^ AHR signaling pathways have also been implicated in *Staphylococcus epidermidis*, *Porphyromonas gingivalis*, *Chlamydia trachomatis*, and *Helicobacter pylori* pathogenesis.^415^, ^416^, ^417^, ^418^, ^419^

The IDO1-AHR axis plays an important immunomodulatory role in pulmonary paracoccidioidomycosis induced after fungal infection with *Paracoccidioides brasiliensis* in mice.^420^ Commensal flora-derived AHR ligands were reported to protect against intestinal damage caused by *Candida albicans*.^421^ These studies suggest that targeting AHR could be a suitable new treatment option for antibacterial and antifungal therapy.

### G Aryl hydrocarbon receptor as a target for cancer treatment

AHR displays both antitumorigenic and protumorigenic properties depending on the context, cancer type, and ligand.^3^^,^^422^ For some tumor types, AHR stimulates cancer cell proliferation, migration, and survival, whereas for others, AHR inhibits these outcomes.^423^

#### 1 Aryl hydrocarbon receptor agonists as anticancer therapeutics

For some tumor types, AHR activation reduces proliferation and survival by regulating cell cycle progression. In breast cancer cells, treatment with AHR agonists reduced RB phosphorylation, leading to G_1_ cell cycle arrest independently of ER-status.^424^^,^^425^ Activated AHR also interacts with the transcription factor forkhead box A1, which together increase cyclin G2 levels, resulting in G_1_ phase arrest independent of ER signaling.^426^ AHR directly regulates cyclin-dependent kinase inhibitor 1B (CDNK1B; also known as p27^Kip1^) transcription, a tumor suppressor that inhibits cyclin-dependent kinase activity, stopping cell division at the G1/S checkpoint.^427^ CDNK1B and AHR were subsequently shown to directly interact, resulting in reduced AHR-mediated transcription.^428^ AHR promoted proteasomal degradation of ER and androgen receptor, thus reducing hormone-dependent breast and prostate cancer cell growth.^229^^,^^429^ Loss of *Ahr* in a transgenic adenocarcinoma of the mouse prostate model increased the frequency of tumor development, which was suggested to be dependent on increased HIF1A signaling due to reduced sequestration of ARNT by AHR.^178^^,^^430^ AHR loss increased cell invasion of MDA-MB-231 human breast cancer cells in digital microfluidic microgel systems.^431^ Treatment with the AHR agonist, 11-chloro-7H-benzimidazo[2,1-a]benzo[de]iso-quinolin-7-one, suppressed, whereas *AHR* loss increased the aggressiveness of MDA-MB-468 triple negative breast cancer cells.^432^ *Ahr* deficiency increased cancer stem cells and tumor frequency and increased epithelial-to-mesenchymal transition and metastasis in lung cancer models.^433^^,^^434^ Moreover, AHR inhibited transforming growth factor *β* small mothers against decapentaplegic homolog signaling, causing reduced cell proliferation and differentiation in medulloblastoma mouse models.^435^ Treatment with the AHR agonist, tranilast, prevented lung metastasis formation of breast cancer cells in immunodeficient NOD.Cg-*Prkdc*^*scid*^ *Il2rg*^*tm1Wjl*^/SzJ mice.^425^ Together, these findings imply context-, assay-, and ligand-dependent prometastatic and antimetastatic properties of AHR in cancer cells.

AHR activation also protected against diethylnitrosamine-induced mouse liver cancer by increasing DNA damage and apoptosis and decreasing proliferative markers.^436^ Loss of *Ahr* increased PanIN 1 lesion formation by increasing pancreatic inflammation of a spontaneous pancreatic ductal adenocarcinoma (PDAC) mouse model.^437^ Treatment of PDAC cell lines with AHR agonists reduced cell proliferation and invasion,^438^^,^^439^ supporting a tumor suppressive role of AHR in PDAC development and progression.

In colorectal cancer models, dietary supplementation with indole-3-carbinol protected against disease progression in an AHR-dependent manner.^313^ AHR activation suppressed, whereas *Ahr* loss increased intestinal tumor development in Apc(Min/+) mice.^228^ Dietary activation of AHR restored barrier homeostasis and prevented colon tumorigenesis by increasing the levels of E3 ubiquitin ligases ring finger protein 43 and zinc and ring finger 3 resulting in reduced Wnt-*β*-catenin signaling.^312^ In general, for colon cancer AHR acts as a tumor suppressor, and this activity is enhanced by AHR activating compounds.^20^

5F203 (2-(4-amino-3-methylphenyl)-5-fluorobenzothiazole) is an AHR agonist that advanced to clinical trials as Phortress [2-(4-amino-3-methylphenyl)-5-fluorobenzothiazole (5F203) lysylamide dihydrochloride], a water-soluble prodrug. Phortress is hydrolyzed to 5F203 in cancer cells, which binds and activates AHR, leading to increased expression of CYP1A1. CYP1A1-catalyzed biotransformation of 5F203 to electrophilic species in tumor cells, as well as increased reactive oxygen species formation and DNA damage, results in cell death.^440^ Phortress was evaluated in a phase I clinical trial in patients with colon, lung, esophageal and stomach cancers done between 2004 and 2012. Thirty-three percent of the patients (14/42) had stable disease; however, because of reported liver and lung toxicity, as well as poor pharmacokinetics it was not further pursued.^441^

Recently, AHR was found to be essential for the antiproliferative actions of PARP7 inhibition in cancer cells.^442^^,^^443^ The underlying mechanism for the reduced growth varies by cell line, with studies showing that it depends on type I IFN induced apoptosis or altered autophagy.^444^^,^^445^ In addition, a range of cancer cell lines that are insensitive to the antiproliferative effects of PARP7 inhibition with RBN2397 or to AHR agonist treatment with tapinarof or 10-chloro-7H-benzimidazo[2,1-a]benzo[de]iso-quinolin-7-one alone were extremely sensitive to combined PARP7 inhibition and AHR agonist treatment. The synergistic response was dependent on AHR and increased expression of AHR target genes, such as CYP1A1, CYP1B1, CDNK1A, and CDNK1B.^446^ For some hormone-dependent breast and prostate cancer cell lines, combined PARP7 inhibition and AHR activation also increases the proteolytic degradation of ER and AR,^446^ suggesting that this combined treatment approach may be a useful therapeutic strategy for a range of tumor types, including hormone-resistant tumors.

#### 2 Aryl hydrocarbon receptor antagonist treatment to reduce tumor growth and enhance antitumor immunity

In tumors, complex cellular and paracrine interactions exist among cancer cells, cancer-associated fibroblasts, tumor-infiltrating lymphocytes, and TAMs, which together with other cells form the TME. The composition and inflammatory state of the TME influences cancer initiation, progression, metastasis, and responses to therapy. Immunotherapy, a type of cancer therapy that targets the immune system, has recently shown unprecedented clinical outcomes. One of the most studied types of treatments is the inhibition of the immune checkpoint proteins, programmed death receptor 1 (PD1), and its ligand, PDL1. In recent years, combination therapies targeting immune checkpoints and innate immunity have emerged as exciting strategies to inhibit tumor development and progression.^447^^,^^448^

AHR is overexpressed and constitutively activated in several cancer types, supporting AHR as a driver of tumorigenesis.^449^^,^^450^ Moreover, AHR plays important roles in multiple stages of tumorigenesis, including cell proliferation, angiogenesis, tissue invasion, tumor-associated inflammation, and metastasis.^19^ AHR promoted the survival and growth of breast cancer cells via reactive oxygen species-dependent AHR activation, resulting in a protumorigenic microenvironment.^234^ Constitutively active AHR transgenic mice develop stomach tumors, supporting the oncogenic potential of the AHR.^451^ Inhibition of AHR improved sensitivity of several PDAC cell lines to the chemotherapeutic gemcitabine.^452^ Mice lacking *Cyp1a1*/*Cyp1a2*/*Cyp1b1* or treatment of wild-type mice with an AHR antagonist reversed the adverse effects of AHR activation and protected against pancreatitis development.^453^ Furthermore, emerging studies provide strong support for a protumorigenic effect of endogenous AHR ligands derived from tryptophan metabolism by IDO1 and TDO. Tumor-derived kynurenine was identified to activate AHR in an autocrine and paracrine manner, resulting in increased tumor growth of glioma cells by suppressing the immune system.^137^ IDO1 and TDO are expressed in many tumors, and their overexpression in preclinical tumor models promotes resistance to immune-based therapies.^454^^,^^455^ However, the highly selective IDO1 inhibitor, epacadostat, failed to achieve efficacy in clinical trials.^456^ This could have been due to the continued generation of tumor kynurenine by TDO or other AHR-activating metabolites generated by IL4I1, which has led to the development of dual IDO1/TDO inhibitors and IL4I1 inhibitors.^457^^,^^458^ AHR activation in tumors from tryptophan metabolites increased the recruitment of immunosuppressive TAMs, which suppress T cell proliferation and induce T cell exhaustion, favoring an immunosuppressive TME that inhibits antitumor immunity.^278^^,^^279^^,^^459^^,^^460^ AHR also upregulated IDO1 levels, leading to increased kynurenine levels to increase tumorigenicity.^461^ Tumor-produced kynurenine also enters CD8^+^ T cells, resulting in AHR activation and the upregulation of PD1 levels.^278^ The increased PD1/PDL1 levels inhibit activated T cells, but this immunosuppression can be overcome with checkpoint inhibitor treatment. Thus, inhibition of AHR is expected to reduce tumor growth and release the “brake” that cancer uses to evade the immune system.

BAY2416964, developed by Bayer, and IK-175, developed by Ikena Oncology, are 2 highly selective AHR antagonists that are currently being investigated in clinical trials as mono-therapy and in combination with anti-PD1 checkpoint inhibition therapy in patients with advanced cancers.^111^^,^^112^ BAY2416964 and IK-175 bind AHR and inhibit AHR activation by endogenous and exogenous agonists.^462^^,^^463^ In a preclinical mouse melanoma model, oral administration of BAY2416964 decreased tumor growth by increasing immune cell infiltration, favoring a proinflammatory TME.^462^ Treatment with BAY2416964 enhanced proinflammatory polarization of human monocytes, potentiated anti-PD1-induced T cell activation and enhanced interferon gamma production of naïve CD4^+^ and CD8^+^ T cells.^462^ In a phase 1 clinical trial (NCT04069026), BAY2416964 was well tolerated, and biomarker analysis provided evidence of target engagement and modulation of immune functions.^464^ However, only one-third of the 67 patients evaluated had stable disease, and no other patients achieved a partial or complete response to BAY2416964.^465^ Driven by these findings, BAY2416964 was investigated in another phase 1 clinical trial (NCT04999202) to determine if BAY2416964 in combination with the anti-PD1 inhibitor, pembrolizumab, was more effective than either treatment alone. The BAY2416964/pembrolizumab trial is listed as terminated/withdrawn. The reason for this is unknown to the authors at the time of the publication of this review.

IK-175 is an AHR antagonist with a favorable ADME (absorption, distribution, metabolism and excretion) and pharmacokinetic profiles in a variety of preclinical species. IK-175 constrained the formation of immunosuppressive T cells in vitro.^463^ In a preclinical CT26 colon cancer mouse model, IK-175 increased M1 polarization of TAMs and CD8^+^ T cells in tumor-draining lymph nodes.^463^ Combination of IK-175 and anti-PD1 antibody reduced colon tumor and IDO1-overexpressing melanoma tumor growth compared with anti-PD1 antibody alone.^463^ IK-175 received a Fast Track Designation from the FDA for patients with advanced urothelial carcinoma.^466^ In support of this, a recent phase 1 trial of IK-175 demonstrated a favorable safety profile, evidence of targeting AHR, and preliminary clinical activity in patients with urothelial carcinoma refractory to prior checkpoint blockade.^467^ As monotherapy, 5 of 13 patients had stable disease, and 1 of 13 had a partial response with IK-175, whereas combination therapy of IK-175 with nivolumab (anti-PD1 therapy) resulted in 14 of 33 patients having stable disease and 2 of 33 showing a partial response.^467^ These data support the further development of AHR inhibitors as antitumor therapeutics to overcome tumor resistance to immune-based therapies.

By contrast, AHR activation by microbial-derived indole-3-aldehyde increased anti-PD1 treatment efficacy and decreased melanoma by increasing the tumor infiltration of CD8^+^ T cells.^468^ In agreement with this, ITE treatment enhanced the ability of anti-PD1 treatment by inhibiting myeloid-derived suppressive cells and promoting CD8^+^ T cell infiltration in a mouse glioblastoma model.^469^ An indole-3-carbinol rich diet increased the efficacy of anti-PD1 therapy in mice injected with fibrosarcoma tumor cells expressing ovalbumin (MCA101-OVA).^470^ Taken together, these findings suggest that the role of AHR in modulating antitumor immunity is cancer type specific. A better understanding of AHR signaling in tumor cells and tumor-associated immune cells is needed to determine how to best modulate AHR to enhance antitumor immunity.

## V Challenges and conclusions

Once considered an unlikely actionable target, AHR has emerged from its origins in toxicology to become a promising clinically relevant target for the treatment of several different diseases. FDA approval of tapinarof confirms the therapeutic potential of AHR modulation and establishes it as a new treatment option in dermatology. The ongoing preclinical development efforts and clinical trials should result in more AHR-targeted therapeutics in the not-so-distant future. As new aspects of AHR biology continually emerge, the interest in pharmacological targeting of AHR will undoubtedly intensify.

Nevertheless, major challenges need to be solved before the therapeutic potential of AHR can be fully realized. AHR’s broad ligand profile, complex signaling roles and context-dependent biological effects complicate ligand specificity, safety, predictability, and clinical efficacy. Overcoming these hurdles will require innovative ligand design, deeper mechanistic insights, improved drug delivery modalities and advanced human-relevant preclinical models. The vast ligand promiscuity, pleiotropic responses, signaling crosstalk and context-dependent actions of AHR might result in a single agent having broad, unpredictable effects across diverse pathways, leading to undesired harmful activity. For example, AHR activation can exhibit anti-inflammatory effects in one context but proinflammatory or toxic responses in another. AHR’s widespread tissue distribution makes it difficult to predict off-target effects and reduce systemic toxicity. The disease context will impact whether activation or inhibition of AHR is the best therapeutic strategy. Designing selective agonists, antagonists, or modulators with predictable, disease-specific outcomes remains a challenging endeavor. Because many AHR ligands are partial agonists, selective modulators, or show biased signaling, a simple agonist/antagonist classification is insufficient. Rapidly metabolized AHR ligands, such as FICZ, ITE, 11-chloro-7H-benzimidazo[2,1-a]benzo[de]iso-quinolin-7-one, and certain plant indoles, activate the AHR but are quickly metabolized, avoiding long-term and systemic toxicity.^471^ In addition, tissue or cell targeted delivery of AHR modulators, such as nanoparticles loaded with agents offer promise in targeting AHR in the desired cell or tissue.^472^ Combining AHR modulators with other therapies, such as checkpoint inhibition for cancer treatment, should permit lower dosing and increase specificity.

AHR is an attractive yet challenging therapeutic target because it regulates a diverse range of signaling pathways in a context-dependent manner. The elucidation of the crystal structure of AHR’s ligand binding domain will help improve the identification of highly selective AHR ligands. The use of human-relevant preclinical models, development of validated biomarkers for target engagement and patient stratification, careful safety evaluation and disease-specific target strategies rather than one-size-fits-all AHR modulation will be necessary for future pharmacological development of AHR ligands.

## Conflict of interest

Jason Matthews is a consultant for NOA Therapeutics. Raitis Bobrovs and Jason Matthews are inventors on patent #W02025114771 issued to the Latvian Institute of Organic Synthesis and The University of Oslo. All other authors declare no conflicts of interest.
